# Identification of pregnancies and their outcomes in healthcare claims data, 2008–2019: An algorithm

**DOI:** 10.1371/journal.pone.0284893

**Published:** 2023-04-24

**Authors:** Elizabeth C. Ailes, Weiming Zhu, Elizabeth A. Clark, Ya-lin A. Huang, Margaret A. Lampe, Athena P. Kourtis, Jennita Reefhuis, Karen W. Hoover

**Affiliations:** 1 National Center on Birth Defects and Developmental Disabilities, Centers for Disease Control and Prevention, Atlanta, Georgia, United States of America; 2 National Center for HIV, Viral Hepatitis, STD, and TB Prevention, Centers for Disease Control and Prevention, Atlanta, Georgia, United States of America; 3 Emory University School of Medicine, Department of Gynecology and Obstetrics, Atlanta, Georgia, United States of America; Kisumu County, KENYA

## Abstract

Pregnancy is a condition of broad interest across many medical and health services research domains, but one not easily identified in healthcare claims data. Our objective was to establish an algorithm to identify pregnant women and their pregnancies in claims data. We identified pregnancy-related diagnosis, procedure, and diagnosis-related group codes, accounting for the transition to International Statistical Classification of Diseases, 10th Revision, Clinical Modification (ICD-10-CM) diagnosis and procedure codes, in health encounter reporting on 10/1/2015. We selected women in Merative MarketScan commercial databases aged 15–49 years with pregnancy-related claims, and their infants, during 2008–2019. Pregnancies, pregnancy outcomes, and gestational ages were assigned using the constellation of service dates, code types, pregnancy outcomes, and linkage to infant records. We describe pregnancy outcomes and gestational ages, as well as maternal age, census region, and health plan type. In a sensitivity analysis, we compared our algorithm-assigned date of last menstrual period (LMP) to fertility procedure-based LMP (date of procedure + 14 days) among women with embryo transfer or insemination procedures. Among 5,812,699 identified pregnancies, most (77.9%) were livebirths, followed by spontaneous abortions (16.2%); 3,274,353 (72.2%) livebirths could be linked to infants. Most pregnancies were among women 25–34 years (59.1%), living in the South (39.1%) and Midwest (22.4%), with large employer-sponsored insurance (52.0%). Outcome distributions were similar across ICD-9 and ICD-10 eras, with some variation in gestational age distribution observed. Sensitivity analyses supported our algorithm’s framework; algorithm- and fertility procedure-derived LMP estimates were within a week of each other (mean difference: -4 days [IQR: -13 to 6 days]; n = 107,870). We have developed an algorithm to identify pregnancies, their gestational age, and outcomes, across ICD-9 and ICD-10 eras using administrative data. This algorithm may be useful to reproductive health researchers investigating a broad range of pregnancy and infant outcomes.

## Introduction

Pregnancy is a condition of broad interest across many medical and health services research domains, but one not easily identified in healthcare claims data as there is typically no marker for pregnancy status, outcome, gestational age, or delivery date. As many pregnancy outcomes (e.g., stillbirth) are rare, large health claims databases (e.g., Merative® MarketScan®, Centers for Medicare and Medicaid Services) represent the most feasible data source to study rare exposures and outcomes. While many algorithms to identify pregnancies in insurance claims data exist [1–8], few use the International Statistical Classification of Diseases, Tenth Revision, Clinical Modification and Procedure Coding Systems (ICD-10-CM/PCS) codes implemented on October 1, 2015 [2, 8]. Algorithms using earlier ICD-9 codes cannot easily be translated to ICD-10 codes. Additionally, ICD-9 algorithms often rely upon “completed weeks of gestation” diagnosis codes (765.XX), predominantly assigned to infant rather than maternal records, necessitating more complex algorithms to estimate gestational age. “Week of gestation” diagnosis codes in the ICD-10 schema (Z3A.XX), assigned to medical visits throughout pregnancy, are likely to allow for improved pregnancy identification, outcome determination, and assessment of gestational age.

We previously developed an algorithm to identify pregnancies in insurance claims data based largely on ICD-9 codes [1]. Herein we describe an updated algorithm that includes ICD-10 codes, as well as additional algorithm enhancements, such as linkage to infant records and a revised hierarchy of pregnancy outcomes. We describe the characteristics of the pregnancy cohort identified in MarketScan Commercial claims data from 2008–2019 using our new comprehensive algorithm to identify pregnancies, their gestational ages, and outcomes across both ICD-9 and ICD-10 coding systems.

## Materials and methods

### Data source

We analyzed 2008–2019 data from the MarketScan Commercial Database. These data include linkable patient-level medical claims from inpatient hospitalizations, outpatient medical visits, and prescriptions dispensed by outpatient pharmacies for a convenience sample of individuals with large employer-sponsored and smaller private health insurance plans (hereafter, “commercial insurance”). Detailed annual enrollment information, including sex and age of the enrollee, type of insurance, information on whether prescription drug information was captured in the data, and duration of enrollment in each calendar year is available. Year, but not date, of birth is available for all enrollees. When covered under the same insurance, family members (e.g., mothers and infants) are linkable. In this analysis, we included women aged 15–49 years with pregnancy-related claims from January 1, 2008 to December 31, 2019 and infants with years of birth from 2008 to 2019, as these were the most recent data available at the time of analysis.

### Pregnancy algorithm

#### Identification of pregnancy-related codes

We used a multi-stage process to identify relevant pregnancy and gestational age codes. First, we identified all diagnosis (ICD-9-CM, ICD-10-CM), procedure (ICD-9-PCS, ICD-10-PCS, current procedural terminology [CPT], and healthcare common procedure coding system [HCPCS]), or diagnosis-related group (DRG) codes related to pregnancy. Second, we bi-directionally mapped all pregnancy-related diagnosis and procedure codes from ICD-9 to ICD-10, as well as from ICD-10 to ICD-9, using the general equivalence mapping tools developed by the Centers for Medicare and Medicaid Services [9]. All pairs from the two crosswalks were de-duplicated. Third, because our algorithm focused on codes indicating a specific pregnancy outcome or gestational age, we focused our broad list on: 1) ICD-9-CM diagnosis, ICD-9, CPT, HCPCS procedure, or DRG codes used in the previous algorithm by Ailes et al. [1], and 2) codes with descriptions that included pregnancy outcome-related terms or phrases (S1 File). Our final code set used for pregnancy identification in maternal claims included maternal codes indicative of a pregnancy outcome, infant birth hospitalization/delivery codes, and specific “weeks of gestation” codes (ICD-9-CM 765.2X codes, ICD-10-CM Z3A.XX codes; full code list provided in S2 File).

We developed additional code sets for use in algorithm verification steps (S2 File). These included codes more broadly indicative of a preterm delivery or prolonged pregnancy, even though a pregnancy outcome could not be assigned to these codes; ectopic pregnancy procedures or methotrexate prescriptions codes; and embryo transfer or insemination codes. All codes and their descriptions were independently reviewed by two co-authors with expertise in obstetrics to determine which pregnancy outcome (live birth, stillbirth, multiple birth of live birth and stillbirth, spontaneous abortion, induced abortion, ectopic pregnancy, or unknown outcome type) and/or gestational age (in weeks) could be assigned or updated from those assigned by Ailes et al. [1]. Discrepancies were resolved via discussion.

#### Pregnancy identification

Annual enrollment files were used to identify women aged 15–49 years and infants (defined as enrollees with birth year equal to enrollment year) during 2008–2019. Among these women, we extracted all inpatient, outpatient, and facility header claims with ≥1 pregnancy- or delivery-related diagnosis, procedure, and/or DRG code from 2007–2019 (2007 was included to more accurately estimate pregnancies ending in 2008). Infant codes recorded on “maternal” claims were captured. Pregnancy identification was based primarily on maternal claims, though infant claims were used during outcome and gestational age verification. In separate datasets, we extracted claims with codes used in outcome and gestational age refinement steps or sensitivity analyses for these same women and infants.

For each woman, we extracted and de-duplicated claims into pregnancy-related ‘records’: distinct combinations of service dates and pregnancy-related codes (diagnosis, procedure, or DRG). We assigned the algorithm-estimated pregnancy outcome and/or gestational age, with the service date serving as proxy for the end of pregnancy/delivery date. Date of last menstrual period (LMP) was assigned by multiplying the algorithm-estimated gestational age by 7 (days per week) and subtracting that number of days from the service date. We also retained the code type (diagnosis, procedure, or DRG) and code version (ICD-9, ICD-10).

To identify records likely belonging to the same pregnancy, we required ≥120 days from any live birth record (including a live birth and stillbirth), and ≥42 days from the end of all other outcomes to the service date and inferred LMP of the subsequent pregnancy. These gaps were chosen because: 1) they were physiologically plausible and 2) exploratory analyses showed that they appeared to differentiate two obvious spikes in the distribution of days between the end of one pregnancy and the estimated LMP of the subsequent pregnancy. In the rare instances when a maternal pregnancy record was not assigned a gestational age estimate (n = 368,234 / 40,724,108, 0.90% records), for the sake of grouping claims into pregnancies only, we assigned a temporary gestational age of 20 weeks (for records associated with live birth or stillbirth outcomes) or 6 weeks (for other pregnancy outcomes). For each pregnancy episode, we identified a pregnancy series (e.g., first, second, third) to account for multiple pregnancies in the same women; minimum and maximum date of service and estimated LMPs for each pregnancy; and indicator variables accounting for type of codes and pregnancy outcomes (e.g., live birth diagnosis code, spontaneous abortion DRG code, etc.).

#### Pregnancy outcome and gestational age verification

To aid in pregnancy outcome estimation, we attempted to link infants born from 2008–2019 to all women with a pregnancy-related record, regardless of initial pregnancy outcome(s), based upon the unique family identifier. Among infants with infant birth hospitalization/delivery codes or preterm/prolonged gestation codes during the year of birth, we required the service date of the earliest infant claim to occur between 7 days before the minimum, and 30 days after the maximum, pregnancy end dates of a linked maternal pregnancy episode, similar to MacDonald et al. [7]. Among infants with 2008–2019 years of birth, but no pregnancy or infant birth hospitalization code that matched the pregnancy episode, we required the year of birth to match the delivery year on the maternal record. If one infant linked to multiple pregnancies, we selected the earliest pregnancy with a live birth code.

We used a hierarchy of outcomes based on code type, pregnancy outcome type, and linkage of a pregnancy episode to an infant to assign our initial pregnancy outcome (Table 1). We identified the earliest pregnancy record of the hierarchy-assigned initial outcome type. This record served as our initial best estimate of pregnancy outcome, end date, gestational age, and LMP. Our hierarchy was revised from our previous analysis [1] and chosen based on exploratory analyses and assumptions, primarily that: 1) identification of a linked infant record is strong evidence that the pregnancy ended in a live birth; 2) diagnosis codes were some of the strongest evidence of a particular pregnancy outcome; 3) pregnancies that included both a spontaneous and induced abortion code were likely to be spontaneous abortions; and, 4) DRG codes for spontaneous abortion, induced abortion, and ectopic pregnancy were stronger evidence than procedure codes; however, DRG codes for live birth were non-specific unless they were the sole pregnancy-related code present. Of note, no DRG code is specific to stillbirth.

**Table 1:** Pregnancy algorithm hierarchy of initial pregnancy outcome, based on outcome type and code type present (Diagnosis, Procedure, or Diagnosis-Related Group [DRG]).

Order	Code type	Pregnancy outcome
1	Diagnosis	Multiple birth including live birth(s) and stillbirth(s)
2	Linkage to infant record	Live birth
3	Diagnosis	Stillbirth
4	Diagnosis	Live birth
5	Diagnosis	Spontaneous abortion
6	Diagnosis	Induced abortion
7	Diagnosis	Ectopic pregnancy
8	DRG	Spontaneous abortion
9	DRG	Induced abortion
10	DRG	Ectopic pregnancy
11	Procedure	Stillbirth
12	Procedure	Spontaneous abortion
13	Procedure	Induced abortion
14	Procedure	Ectopic pregnancy
15	Procedure	Live birth
16	DRG	Live birth

Additional refinements were made (see decision algorithms in S3 File), to adjust the final pregnancy outcome for a small proportion of pregnancies (1.3%, 84,679/6,520,768, S1 Table). These included recoding some pregnancies initially identified as induced abortions to ectopic pregnancies; requiring ectopic pregnancies to have a proximate ectopic pregnancy procedure or methotrexate prescription, similar to the methods of Hoover et al. [10] and Sarayani et al. [8]; and modifying pregnancies initially coded as stillbirths to other outcomes, based on available gestational age estimates and co-occurring spontaneous abortion, induced abortion, or live birth records.

After finalizing pregnancy outcomes, we made additional refinements to gestational age estimates, as described in more detail in S3 File. These modifications were based on the last ‘direct’ gestational age code estimates (e.g., ICD-10-CM Z3A.## or ICD-9-CM: 765.XX codes, S2 File) available for the pregnancy episode, as well as the presence of codes indicating a preterm or prolonged pregnancy either on the maternal or linked infant record. The LMP estimate from the selected direct gestational age claim, when available, was used as the final LMP estimate and was used to re-calculate the gestational age at the end of pregnancy by subtracting the final LMP from the pregnancy end date and dividing that total by 7 (days per week).

**Fig 1:**
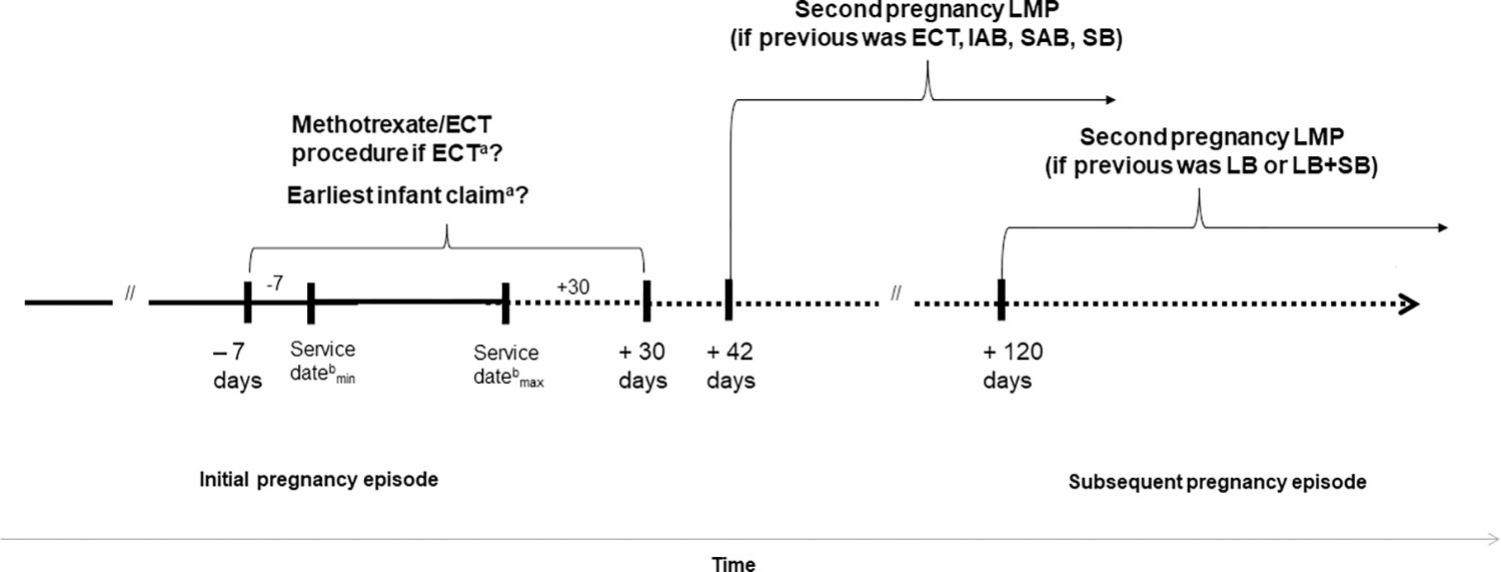
Schematic of select pregnancy algorithm implementation steps. Abbreviations: ECT = ectopic pregnancy, IAB = induced abortion, LB = live birth, LMP = last menstrual period, SAB = spontaneous abortion, SB = stillbirth. ^a^ Select verification steps for pregnancy outcome type and/or gestational age. ^b^ Represent the minimum and maximum service dates associated with claims that have a pregnancy outcome diagnosis, procedure, and/or DRG code. See supporting files for more information.

A small number of pregnancies with missing outcome (5.7%, S1 Table) remained at the end of this process. Pregnancy outcome could have been missing due to lapses in insurance enrollment at the end of pregnancy or because pregnancies were ongoing as of December 31, 2019 (the most recent data available at the time of the analysis). To address this, we assumed all pregnancies with missing outcomes were live births at 39 weeks gestation and estimated their date of delivery as their maximum pregnancy episode LMP + (39 weeks x 7 days/week). If this date was after December 31, 2019, we considered their pregnancy outcome to be right censored and unobservable. If the estimated pregnancy end date occurred before December 31, 2019, but after a woman’s last month and year of insurance enrollment (as identified using the annual enrollment files), we also considered the pregnancy outcome to be unobservable and excluded these pregnancies from analysis. A simplified schematic of the pregnancy algorithm steps is shown in Fig 1.

### Analyses

We stratified characteristics of women and their pregnancies in our cohort by ICD “era” (1/1/2008-9/30/2015 deliveries for ICD-9 compared to 10/1/2015-12/31/2019 for ICD-10 deliveries). Overall and for each stratum, we estimated the total and average number of pregnancies per woman, average number of pregnancy- or gestational age-related records per pregnancy, and the distribution of pregnancies by outcome, delivery year, gestational age, maternal age at delivery, U.S. Census region, type of insurance, and continuous enrollment (i.e., at least one day of enrollment per month with no more than a two month gap in enrollment) before/during pregnancy. While the MarketScan Commercial data represent a convenience sample of persons with commercial insurance, we applied weights to generate national estimates among women with commercial insurance, derived from the American Community Survey [11]. To better understand the potential impact of missing prenatal exposures on studies of infant outcomes, we compared the aforementioned characteristics between pregnancies that could be linked (vs. not) to infant records. We also described the proportion of infant linked live births with any pregnancy-related claim available.

Lastly, we conducted two sensitivity analyses. To assess the potential impact of the specific weeks of gestation codes on our gestational age estimation, we removed these codes and calculated the gestational age using the remaining information available. We also conducted a sensitivity analysis, similar to the study of Bird et al. [12], among the small subset of pregnancies with embryo transfer or insemination codes. The service date of these procedures approximates the date of conception, which typically occurs 14 days after LMP. Because women might have had multiple unsuccessful fertility procedures prior to a successful one but also to allow for some inaccuracies in our LMP estimates, we identified the last fertility procedure that occurred from 56 days before the pregnancy’s estimated LMP through the end of pregnancy. We compared the LMP based on fertility procedure date (fertility procedure service date– 14 days) to the LMP based on our final algorithm.

MarketScan data are collected as part of billing for routine patient care and deidentified before access is granted to researchers; therefore no Institutional Review Board review was needed. All analyses were conducted using SAS v9.4 (Cary, NC; SAS code available in S1 Data). Because of the large sample size very small differences between groups could be considered statistically significant, we chose not to conduct statistical testing, but rather considered any differences of ≥5% between groups to be notable.

## Results

**Fig 2:**
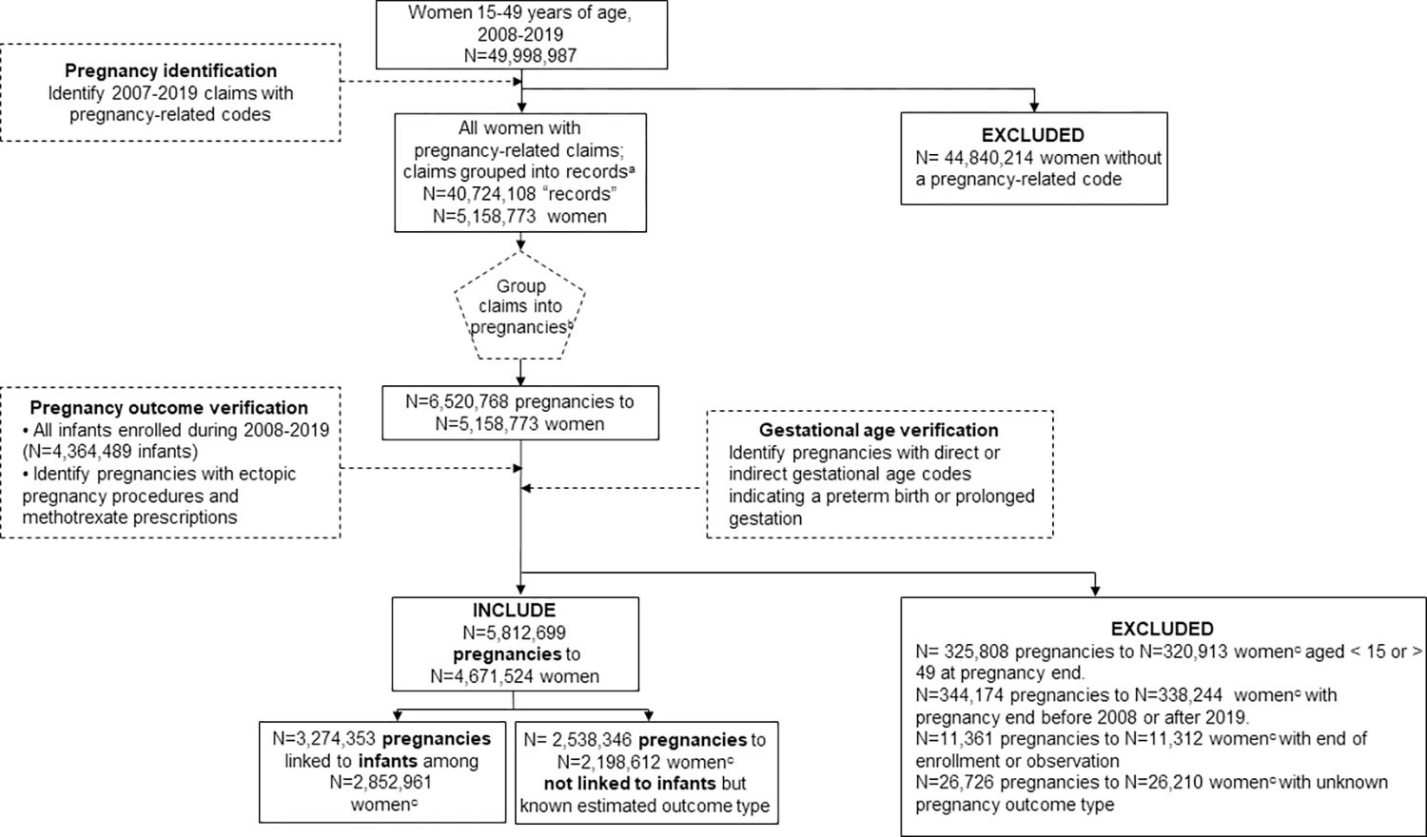
Identification of cohort of pregnant women, MarketScan commercial data, 2008–2019. ^a^ Extracted and then deduplicated inpatient, outpatient, and facility header claims with codes from S2 into pregnancy-related ‘records’: distinct combinations of service dates and pregnancy-related codes (diagnosis, procedure, or diagnosis related group). ^b^ Records likely belonging to the same pregnancy were grouped together into pregnancy episodes. ^c^ Because women could have more than one pregnancy, the sum of these categories will be greater than the total number of women.

Among the 49,998,987 women aged 15–49 years during 2008–2019 in the MarketScan Commercial data, we identified 40,724,108 unique pregnancy-related records in 5,158,773 (10.3%) women (Fig 2). Collapsing records into pregnancy episodes resulted in a total of 6,520,768 possible pregnancies. During outcome verification, we also identified 4,364,489 infants born during 2008–2019, of which 3,274,353 were linked to potential pregnancies. We found consistency in most recorded pregnancy outcomes within pregnancy episodes, with the exception that stillbirth claims were rarely (<10%) the only type of pregnancy outcome in a pregnancy episode (S2 Table), and often occurred in combination with live birth or spontaneous abortion records. Among the 57,277 pregnancies we initially coded as stillbirths, 34,467 (60.2%) remained after verification (S1 Table). Among 85,410 pregnancies initially coded as ectopic pregnancies, 42,275 (49.5%) were verified using ectopic procedure or methotrexate prescription codes (S1 Table). At the end of the verification process, 708,069 pregnancies had a missing pregnancy outcome or were outside our delivery years or ages of interest and were excluded (Fig 2). A total of 5,812,699 pregnancies to 4,671,524 women were included in our final cohort (Fig 2).

Most (n = 4,401,015, 75.7%) pregnancies in the final cohort ended in the ICD-9 era between January 1, 2008 and September 30, 2015 (Table 2). The mean number of pregnancies per woman was 1.2 across all years (1.2 in the ICD-9 era, 1.1 in the ICD-10 era). There were an average of 6.5 (interquartile range [IQR]: 3–8) pregnancy-related records per pregnancy across the entire cohort, though pregnancies delivered in the ICD-10 era had more records (mean: 10.6, IQR: 6–14) than those in the ICD-9 era (mean: 5.2, IQR: 3–6). Over 85% of pregnancies from the ICD-10 era had at least one direct gestational age code, while many fewer (1.3%) did during the ICD-9 era.

**Table 2:** Characteristics of pregnancy cohort overall and by international statistical classification of diseases clinical modification and procedure coding systems (ICD) era MarketScan commercial data, 2008–2019 (N = 5,812,699 pregnancies).

Characteristic	ICD-9 “era” (1/1/2008 to 9/30/2015)		ICD-10 “era” (10/1/2015 to 12/31/2019)		Total		Weighted^a^	
	N	(%)	N	(%)	N	(%)	N	(%)
Number of women	3,626,282		1,241,246		4,671,524		N/A	
Number of pregnancies	4,401,015		1,411,684		5,812,699		24,878,171	
Mean (IQR) pregnancies/woman	1.2 (1,1)		1.1 (1,1)		1.2 (1,1)		N/A	
Number of pregnancy-related records	22,977,495		15,030,686		38,008,181		N/A	
Mean (IQR) pregnancy-related records	5.2 (3,6)		10.6 (6,14)		6.5 (3,8)		N/A	
> = 1 gestational-age specific code^b^	57,452	1.3	1,221,441	86.5	1,278,893	22.0	7,970,655	32.0
Pregnancy outcome								
Livebirth	3,406,957	77.4	1,120,780	79.4	4,527,737	77.9	19,574,369	78.7
Spontaneous abortion	720,483	16.4	220,082	15.6	940,565	16.2	3,922,049	15.8
Induced abortion	212,558	4.8	51,312	3.6	263,870	4.5	1,057,551	4.3
Ectopic pregnancy	33,037	0.8	9,235	0.7	42,272	0.7	166,351	0.7
Stillbirth	22,753	0.5	9,609	0.7	32,362	0.6	134,955	0.5
Livebirth + stillbirth	5,227	0.1	666	0.1	5,893	0.1	22,897	0.1
Delivery year								
2008	627,317	14.3	-	-	627,317	10.8	2,134,115	8.6
2009	515,382	11.7	-	-	515,382	8.9	2,129,850	8.6
2010	552,127	12.5	-	-	552,127	9.5	1,961,210	7.9
2011	641,085	14.6	-	-	641,085	11.0	1,983,580	8.0
2012	658,503	15.0	-	-	658,503	11.3	1,994,599	8.0
2013	550,283	12.5	-	-	550,283	9.5	1,996,363	8.0
2014	584,020	13.3	-	-	584,020	10.1	1,990,946	8.0
2015	272,298	6.2	86,789	6.1	359,087	6.2	2,240,760	9.0
2016	-	-	352,379	25.0	352,379	6.1	2,087,758	8.4
2017	-	-	327,065	23.2	327,065	5.6	2,114,515	8.5
2018	-	-	337,427	23.9	337,427	5.8	2,114,627	8.5
2019	-	-	308,024	21.8	308,024	5.3	2,129,848	8.6
Gestational age (weeks)								
8–10	583,923	13.3	183,298	13.0	767,221	13.2	3,173,501	12.8
11–19	373,109	8.5	91,601	6.5	464,710	8.0	1,902,977	7.6
20–23	2,169	0.05	3,533	0.3	5,702	0.1	29,024	0.1
24–28 (early preterm)	11,843	0.3	7,556	0.5	19,399	0.3	87,578	0.4
29–33 (moderate preterm)	53,196	1.2	25,379	1.8	78,575	1.4	347,449	1.4
34–36 (late preterm)	169,937	3.9	70,581	5.0	240,518	412	1,068,219	4.3
37–38 (early term)	1,254	0.03	238,584	16.9	239,838	4.1	1,498,738	6.0
39–41 (term)	3,135,364	71.2	736,655	52.2	3,872,019	66.6	16,121,861	64.8
≥ 42 (post-term)	57,649	1.3	49,199	3.5	106,848	1.8	569,814	2.3
Unknown	12,571	0.3	5,298	0.4	17,869	0.3	79,008	0.3
Maternal age at delivery (years)								
15–19	131,218	3.0	21,319	1.5	152,537	2.6	536,993	2.2
20–24	533,132	12.1	160,463	11.4	693,595	11.9	2,610,744	10.5
25–29	1,142,938	26.0	345,885	24.5	1,488,823	25.6	6,392,576	25.7
30–34	1,457,383	33.1	487,683	34.6	1,945,066	33.5	8,608,510	34.6
35–39	848,159	19.3	303,562	21.5	1,151,721	19.8	5,081,918	20.4
40–44	258,250	5.9	83,087	5.9	341,337	5.9	1,476,117	5.9
45–49	29,935	0.7	9,685	0.7	39,620	0.7	171,313	0.7
Census region								
Northeast	783,919	17.8	252,665	17.9	1,036,584	17.8	4,967,527	20.0
Midwest	1,018,340	23.1	284,615	20.2	1,302,955	22.4	5,710,424	23.0
South	1,647,726	37.4	627,085	44.4	2,274,811	39.1	8,316,214	33.4
West	863,035	19.6	244,717	17.3	1,107,752	19.1	5,792,398	23.3
Unknown	87,995	2.0	2,602	0.2	90,597	1.6	91,607	0.4
Type of insurance^c^								
Large employer	1,966,850	44.7	1,054,515	74.7	3,021,365	52.0	15,111,915	60.7
Small health plan	2,434,074	55.3	357,142	25.3	2,791,216	48.0	9,766,138	39.3
Continuous enrollment								
LMP through the end of pregnancy	3,239,542	73.6	1,115,181	79.0	4,354,723	74.9	18,633,350	74.9
Not continuously enrolled	1,161,473	26.4	296,503	21.0	1,457,976	25.1	6,244,820	25.1

IQR: Interquartile range; LMP: Last menstrual period; N/A: Not applicable because some women had pregnancies in both ICD-9 and ICD-10 eras and weighted estimates are based on the woman, not pregnancy. One woman also had different weights in different years.

^a^ Estimated number and % of people with commercial insurance

^b^ Defined as ≥ 1 claim with a direct gestational age code (see Supporting files)

^c^ n = 118 missing health plan type

Of the 5,812,699 identified pregnancies, the majority (77.9%) were livebirths, followed by spontaneous abortion (16.2%); outcome distributions were similar between ICD-9 and ICD-10 eras (Table 2). While the unweighted annual number of identified pregnancies was smaller in later years because of changes in the MarketScan data contributors since 2015, weighted estimates remained stable. A higher proportion of ICD-9 era pregnancies (71.2%) were estimated to end at term (39–41 weeks) compared to ICD-10 pregnancies (52.2%); fewer early term (37–38 weeks) and fewer post-term (≥42 weeks) pregnancies were estimated in the ICD-9 era compared to the ICD-10 era (0.03% vs. 16.9% and 1.3% vs. 3.5%, respectively). Among live births, weighted frequencies of preterm birth were lower in the ICD-9 era than the ICD-10 era (6.7 vs. 8.8%; S3 Table) as were post-term (≥42 weeks) birth estimates (1.7% vs. 4.8%). Overall, the majority of pregnant women with commercial insurance were 25–34 years of age (59.1%), were South (39.1%) or Midwest (22.2%) residents had large employer-sponsored insurance (52.0%) and had continuous enrollment during pregnancy (74.9%).

In the final cohort, 4,533,630 pregnancies ended in a live birth or livebirth and stillbirth, of which 3,274,353 (72.2%) were linked to infant records in the database (Fig 2, Table 3). Among live births linked to an infant, infant birth hospitalization codes were found on the infant record more frequently than on the linked maternal record (86.2% vs 33.7%). Delivery year, type of insurance, and proportion with continuous enrollment were similar between women with live birth pregnancies that linked to an infant compared to those that did not (Table 3). However, live birth pregnancies that did not link to an infant were more often estimated to end at term (88.9% vs. 83.7%). Additionally, women with pregnancies that did not link to an infant were younger (15–24 years at delivery) compared to those with a linked infant (5.9% vs. 34.5%), more likely to reside in the South (45.5% vs. 37.9%) and less likely to reside in the Northeast (12.9% vs. 17.6%), and more likely to be a child of the primary insurance holder (30.7% vs. 0.9%).

**Table 3:** Comparison of live birth pregnancies that linked and did not link to an infant record, Marketscan commercial data, 2008–2019 (N = 4,533,630).

Characteristic	Live Births Linked to Infants (N = 3,274,353 pregnancies)		Live Births Not Linked to Infants (N = 1,259,277 pregnancies)	
	N	(%)^a^	N	(%)^a^
Pregnancy outcome				
Livebirth	3,270,642	99.9	1,257,095	99.8
Livebirth + stillbirth	3,711	0.1	2,182	0.2
Infant birth hospitalization claim available^b^				
Any	3,035,057	92.7	437,334	34.7
On infant record	2,821,668	86.2	N/A	
On maternal record	1,102,876	33.7	437,334	34.7
Delivery year				
2008	364,233	11.1	115,945	9.2
2009	305,034	9.3	93,835	7.5
2010	311,472	9.5	115,513	9.2
2011	358,469	11.0	139,452	11.1
2012	367,621	11.2	144,428	11.5
2013	304,246	9.3	121,953	9.7
2014	320,216	9.8	134,432	10.7
2015	195,993	6.0	88,194	7.0
2016	196,463	6.0	84,924	6.7
2017	184,339	5.6	76,673	6.1
2018	193,730	5.9	74,721	5.9
2019	172,537	5.3	69,207	5.5
Gestational age (weeks)				
20–23	2,328	0.1	1,426	0.1
24–28 (early preterm)	14,903	0.5	3,801	0.3
29–33 (moderate preterm)	56,216	1.7	8,841	0.7
34–36 (late preterm)	201,951	6.2	38,014	3.0
37–38 (early term)	163,230	5.0	76,363	6.1
39–41 (term)	2,739,662	83.7	1,120,074	88.9
≥ 42 (post-term)	96,063	2.9	10,758	0.9
Maternal age at delivery (years)				
15–19	10,960	0.3	93,353	7.4
20–24	184,633	5.6	341,040	27.1
25–29	876,232	26.8	335,228	26.6
30–34	1,298,769	39.7	291,240	23.1
35–39	722,704	22.1	151,124	12.0
40–44	168,490	5.2	39,665	3.2
45–49	12,565	0.4	7,627	0.6
Census region				
Northeast	575,959	17.6	162,731	12.9
Midwest	772,282	23.6	276,852	22.0
South	1,240,038	37.9	573,023	45.5
West	634,086	19.4	227,035	18.0
Unknown	51,988	1.6	19,636	1.6
Maternal relation to primary insurance holder^c^				
Self/Primary	1,555,519	47.5	648,954	51.5
Dependent of primary holder	1,690,910	51.6	223,171	17.7
Child of primary holder	27,918	0.9	387,052	30.7
Maternal Insurance at delivery^c^				
Large employer	1,719,782	52.5	644,623	51.2
Small health plan	1,554,565	47.5	614,554	48.8
Continuous enrollment				
LMP through the end of pregnancy	2,321,853	70.9	902,870	71.7
Not continuously enrolled	952,500	29.1	356,407	28.3

LMP: Last menstrual period

^a^ Column %

^b^ Not mutually exclusive, as infant codes may be found on both mother and infants’ claims

^c^ n = 106 missing health plan information

In our first sensitivity analysis, we noted that use of direct gestational age codes resulted in shifts in the final gestational age categories, particular for pregnancies in the ICD-10 era and for pregnancies that would have been considered as ‘term’ based on other available codes (S4 Table). In our second sensitivity analysis of women who had an embryo transfer or insemination procedure code, we identified 107,870 pregnancies with the procedure occurring between 56 days before LMP through the end of pregnancy. On average, algorithm- and fertility procedure-derived LMP estimates were within a week of each other (mean difference: -4 days, median: -2 days [IQR: -13 to 6 days], S5 Table), with estimates closer among pregnancies estimated to end in a live birth (mean: 1 day, n = 78,283; distribution in S1 Fig) than those estimated to end in a non-live birth (mean: -17 days; n = 29,587).

## Discussion

We identified 5.8 million pregnancies during 2008–2019 in MarketScan Commercial data, but our algorithm is applicable to any administrative or electronic health record data with service date and diagnosis, procedure, or DRG codes, across both ICD-9 and ICD-10 coding schemas. It builds upon previous algorithms [1, 3, 6–8, 10, 12–18] to include components proposed separately but rarely combined in one algorithm (e.g., linkage to infants, verification of ectopic pregnancies, ICD-10 and ICD-9 codes). Furthermore, our algorithm carefully assigned pregnancy outcome when multiple outcome codes were present.

While we were unable to externally validate our algorithm, comparisons of our weighted estimates to national data and sensitivity analyses support our algorithm’s framework. Our observed stillbirth prevalence (8.0 per 1,000 live births) fell within estimates using fetal death certificates alone (5.9 per 1,000 live births [19]) and in combination with stillbirth surveillance (10.0 per 1,000 live births plus stillbirths [20]). Our sensitivity analysis among women with fertility procedures showed good agreement with algorithm estimates, proving further reassurance of our algorithm’s accuracy.

Despite inclusion of specific weeks of gestation and broader ‘preterm’ codes, our weighted ICD-9 era preterm birth rate (6.7%) was lower than contemporaneous national estimates based on obstetric estimates (9.7% [21]; S3 Table), but our ICD-10 era rate was closer (8.8% vs. 10.0%). Notably, our analysis did not include women with Medicaid insurance, who may experience an increased frequency of preterm births [22], and national estimates based on LMP (similar to our method) tend to have higher post-term (and preterm) estimates [23].

By linking to infant records, we internally validated 72% of live births; the remaining may not have linked because infants were on other insurance plans (e.g., their fathers’) and younger mothers tended to be on their parents’ insurance, which typically does not cover the resulting grandchildren. Identification of non-live birth outcomes was more challenging; efforts to improve the coding accuracy of these outcomes in clinical practice could help. Requiring proximate relevant procedures or prescriptions improved the specificity of our ectopic pregnancy ascertainment. We prioritized assignment of spontaneous abortion outcomes over induced abortions if both were present, in contrast to previous algorithms [1, 7, 8], as spontaneous abortions can be treated with procedures also used for induced abortions, and pregnancies among women with fertility procedure codes had both outcome types yet induced abortions are likely less common in this group. However, these decisions might have overestimated the number of spontaneous abortions.

Though relatively rare, identification of pregnancies ending in stillbirth posed challenges. Vital statistics data show a bimodal distribution of the gestational ages of stillbirths (at 20 and 39 weeks), rendering our experts unable to assign one gestational age estimate to some common stillbirth codes. Pregnancies with a stillbirth code also overwhelmingly (91%) had other outcome codes. By examining the timing and distribution of other outcome and gestational age codes in these pregnancies, and making subsequent adjustments to the final pregnancy outcome, our stillbirth prevalence was in the range of published estimates [19, 20].

Overall, our large sample size allowed for identification of rare pregnancy outcomes. Inclusion of non-live birth outcomes was a critical component of our algorithm, as restricting to live births can lead to selection bias in epidemiologic studies [24–26]. Our comparison of ICD-9 to ICD-10 eras suggests that more detailed gestational age estimation is possible in the latter time period.

Limitations of our approach include that billing data are not designed for scientific investigations and their use may result in misclassification of some pregnancy outcomes or gestational ages because of billing errors, “rule-out” diagnoses, and other factors. While use of both ICD-9 and ICD-10 coding schema was a strength of our approach, and we, and others [8], have explored the impact of these changes, use of both coding systems could have led to coding errors. Additionally, we made assumptions about the gestational age at which many pregnancy outcomes occurred and were unable to verify our algorithm estimates compared to medical or birth records, though our sensitivity analysis and comparison to national estimates provides confidence in our algorithm. Lastly, we lacked information on healthcare experiences not covered by insurance.

These limitations notwithstanding, our algorithm represents a methodological advance in use of information from administrative data and could be useful for researchers, public health practitioners, health systems, third-party payers, and others to answer questions about pregnant women and maternal and infant outcomes, including rare outcomes. Internally validated algorithms like ours can have broad applications to clinical research. Additionally, use of standardized pregnancy algorithms will facilitate comparisons across different studies.

## Supporting information

S1 File List of pregnancy outcome-related terms or phrases-search terms. (DOCX)

S2 File List of diagnosis, procedure, and diagnosis-related group codes used in algorithm. (XLSX)

S3 File Decision algorithms. (DOCX)

S1 Data SAS programming package. (ZIP)

S1 Table Initial pregnancy outcome compared to final pregnancy outcome, MarketScan 2008–2019 (N = 6,520,768 potential pregnancies). (DOCX)

S2 Table Frequency of single pregnancy outcome type in pregnancy episodes, by final pregnancy outcome type, MarketScan 2008–2019 (N = 5,812,699 pregnancies). (DOCX)

S3 Table Gestational age distribution of pregnancies estimated to end in a live birth (weighted total: 19,190,432 pregnancies^a^) to National Vital Statistics System^b^ (NVSS) estimates. (DOCX)

S4 Table Sensitivity analysis comparing estimated gestational age without use of direct gestational age codes to the final algorithm estimated gestational age using direct gestational age codes, by gestational age group and International Statistical Classification of Diseases, Clinical Modification and Procedure Coding System (ICD) era. (DOCX)

S5 Table Sensitivity analysis of difference between algorithm-estimated last menstrual period (LMP) and fertility (embryo transfer or insemination) procedure-based LMP estimate^a^, by pregnancy outcome type and ICD era, among pregnancies with co-occurring assisted reproductive procedures (n = 107,870 pregnancies^b^). (DOCX)

S1 Fig Sensitivity analysis: Distribution of difference between algorithm-estimated last menstrual period (LMP) and fertility (embryo transfer or insemination) procedure-based LMP estimate^a^, among pregnancies estimated to end in a live birth with co-occurring assisted reproductive procedures (n = 73,241 pregnancies^b^). (DOCX)
