# Supplementary material for: Identification of pregnancies and their outcomes in healthcare claims data, 2008–2019: An algorithm
Source: PLoS One. 2023 Apr 24;18(4):e0284893. doi: 10.1371/journal.pone.0284893 (PMC10124843; doi:10.1371/journal.pone.0284893)
Supplement: S1 File — (DOCX) [file pone.0284893.s001.docx]

**S1 File. List of pregnancy outcome-related terms or phrases-search terms.**

- “abortion”
- “birth”
- “born”
- “conception”
- “delivery”
- “death”
- “ectopic
- “induced abortion"
- “liveborn”
- “live birth”
- “miscarriage”
- “missed abortion”
- “pregnancy”
- “still”[birth/born],
- “spontaneous”[abortion]
- “threatened”[abortion]
