# Supplementary material for: Identification of pregnancies and their outcomes in healthcare claims data, 2008–2019: An algorithm
Source: PLoS One. 2023 Apr 24;18(4):e0284893. doi: 10.1371/journal.pone.0284893 (PMC10124843; doi:10.1371/journal.pone.0284893)
Supplement: S3 File — (DOCX) [file pone.0284893.s003.docx]

**S3 File. Decision algorithms**

1. **Revising pregnancy outcome assignment**

After assigning the initial pregnancy outcome, based on the hierarchy described in the Methods, we made additional adjustments as follows.

Induced abortions

Induced abortions with missing gestational age (based on gestational age assigned to induced abortion codes) were reclassified as ectopic pregnancies if an ectopic pregnancy code was present in the same pregnancy episode.

Ectopic pregnancies

For further identification of ectopic pregnancies, we used similar methods to Hoover et. al (1) and Sarayani et al. (2). We identified pregnancy episodes with an ectopic procedure or methotrexate administration procedure code (Appendix 2) occurring between 7 days before the first service date and 30 days after the last service date of each pregnancy. Pregnancies initially coded as ectopic but not meeting these criteria were shifted to ‘unknown’ outcome.

Stillbirths

While interim analyses showed that pregnancy episodes for most outcomes were comprised mostly of only one type of outcome, stillbirth claims were infrequently (<10%) the only type of pregnancy outcomes in a pregnancy episode (Appendix 5), and often occurred in combination with live birth or spontaneous abortion claims. In addition, we were unable to assign a gestational age estimate to some of the more commonly occurring stillbirth codes (ICD-9-CM 656.4X for “intrauterine death” and ICD-10-CM O36.4XX# for “maternal care for intrauterine death”) because of the bimodal nature of the gestational age distribution of stillbirths around 20 and 39 weeks gestation observed in vital statistics data (3). Thus, we identified whether stillbirth pregnancies also had a weeks of gestation code, as described in section (B) below and updated the stillbirth gestational age estimate using those direct gestational age codes.

We re-examined stillbirth pregnancies with missing or implausible (< 20 weeks) gestational age estimates, and identified whether there were any spontaneous abortion, induced abortion, ectopic pregnancy, or live birth pregnancy records that occurred during the same pregnancy episode. If the stillbirth pregnancy episode also had a live birth diagnosis code on or before the stillbirth claim and the live birth code had a gestational age estimate, the pregnancy outcome remained as a stillbirth, but the LMP from the live birth code was applied to the stillbirth pregnancy and its gestational age was updated based on that LMP and date of delivery. If the live birth diagnosis code occurred 14 or more days after the stillbirth, then the stillbirth was reclassified as a live birth, under the assumption that the stillbirth code was a rule-out diagnosis. If a stillbirth pregnancy still had missing/implausible gestational age, we identified whether a weeks of gestation code, as described in section (B) below, was available and updated the stillbirth gestational age estimate using those direct gestational age codes. Lastly, if a stillbirth still had a missing or implausible gestational age but a spontaneous abortion, induced abortion, or ectopic pregnancy code (i.e., diagnosis, procedure, or DRG code) was present during the pregnancy episode, then the stillbirth was reclassified as a spontaneous or induced abortion or ectopic pregnancy, respectively.

1. **Revising gestational age estimates**

The application of ICD-10-CM ‘weeks of gestation’ codes (e.g., Z3A.XX, see Appendix 2) to maternal records throughout a pregnancy provided an opportunity to refine the gestational age at the end of pregnancy and the estimated LMP for pregnancies with claims on/after October 1, 2015. We extracted all claims with a weeks of gestational code from women with pregnancy episodes identified in our algorithm. We selected the last claim with one of these weeks of gestational age codes within each pregnancy episode, along with the associated gestational age and estimated LMP. We only used the last claim because exploratory analyses identified very little variability in the LMP estimates based on claims within a given pregnancy episode. When women had repeated claims with the same gestational age code, we selected the earliest claim with that code, to account for the earliest date with an estimated LMP.

ICD-9-CM and ICD-10-CM codes also existed that provided broader categories of gestational age, such as preterm birth and prolonged pregnancy (Appendix 2). Similar to the approach used by Sarayani [2], we classified these codes as direct or indirect indicators of a preterm or prolonged pregnancy gestation. Direct indicators were those that indicated “preterm”, “post-term”, “prolonged”, or “immaturity” in the code description, while indirect codes were those related to infant birthweight. These decisions were based on previous algorithms (4) and the distribution of gestational ages on birth certification of live births in national vital statistics data (5). We extracted all maternal or linked infant claims with a preterm or prolonged pregnancy code from women with live birth, live birth and stillbirth, or stillbirth pregnancy episodes identified in our algorithm. We selected the last claim from this set of codes, among those that fell between the minimum and maximum pregnancy episode dates, along with the associated gestational age and estimated LMP. After our assignment of pregnancy outcome, occasionally there was no record of the assigned pregnancy outcome type with a gestational age estimate available. For pregnancies estimated to end in spontaneous abortions we used any induced abortion records included in the same pregnancy.

**REFERENCES**

1. Hoover KW, Tao G, Kent CK. Trends in the diagnosis and treatment of ectopic pregnancy in the United States. Obstet Gynecol 2010 Mar;115(3):495-502.

2. Sarayani A, Wang X, Thai TN, Albogami Y, Jeon N, Winterstein AG. Impact of the Transition from ICD-9-CM to ICD-10-CM on the Identification of Pregnancy Episodes in US Health Insurance Claims Data. Clin Epidemiol 2020;12:1129-38.

3. Hoyert DL, Gregory EC. Cause of Fetal Death: Data From the Fetal Death Report, 2014. Natl Vital Stat Rep 2016 Oct;65(7):1-25.

4. Hornbrook MC, Whitlock EP, Berg CJ, Callaghan WM, Bachman DJ, Gold R, et al. Development of an algorithm to identify pregnancy episodes in an integrated health care delivery system. Health Serv Res 2007 Apr;42(2):908-27.

5. Martin JA, Hamilton BE, Osterman MJK, Driscoll AK. Births: Final Data for 2019. Natl Vital Stat Rep 2021;70(2):1-51.
