# Supplementary material for: Identification of pregnancies and their outcomes in healthcare claims data, 2008–2019: An algorithm
Source: PLoS One. 2023 Apr 24;18(4):e0284893. doi: 10.1371/journal.pone.0284893 (PMC10124843; doi:10.1371/journal.pone.0284893)
Supplement: S1 Data — (ZIP) [file pone.0284893.s004.zip › S5. SAS programming package/S5 File. SAS programming package.docx]

Ailes, Zhu et al. Identification of pregnancies and their outcomes in healthcare claims data, 2008-2019: An algorithm

Last Updated: March 22, 2023

**S5 File-- SAS Programming Package**

1. **Getting Started**
2. Identify the location of raw claims data files and determine the file structure (i.e., number of diagnosis, procedure, and/or DRG code variables included; ability to link mothers to infants; etc.).
3. Update the SAS code variable names based on the raw data being used. Because the sample SAS code was written for MarketScan data, the following variables were used:
   - 1. Enrolid=Unique subject ID number*
     2. Svcdate=Service date of claim
     3. DX=Diagnosis code
     4. PROC=procedure code
     5. DRG=DRG code
4. Download the code lists included in the SAS code package (e.g., code_m_pgid.sas7bdat). These should be saved to whichever folder will be assigned to the **pgwork** library. Some interim datasets will be directed here as well.

* A unique family identifier (Famid) was created based on the Enrolid as well.

1. **SAS Code Lists**

Included in the programming package are the following datasets that are used in the SAS program modules. These datasets correspond to the “List of diagnosis, procedure, and diagnosis-related group codes used in algorithm Excel file” (S2 Table).

| **SAS Data File** | **Contents** | **Note** | **Corresponding S2 Table Column** |
| --- | --- | --- | --- |
| Code_m_pgid | Combined code sets for pregnancy episode identification | Diagnosis/Procedure/DRG codes with assigned pregnancy outcome and gestational age | Pregnancy identification |
| Code_ectproc | Procedure codes associated with ectopic pregnancy | Code version and code | Verification of ectopic pregnancies (procedures) |
| Code_metho | National Drug Codes (NDC) codes for Methotrexate to verify ectopic pregnancy |  | Verification of ectopic pregnancies (medications) |
| Code_infant | Diagnosis codes used for infants’ conditions | Diagnosis codes with assigned pregnancy outcome and gestational age | Infant verification |
| Code_preterm_prolong | Diagnosis codes used for preterm birth and prolonged pregnancy verification |  | Preterm/Post-term gestational age verification |

1. **Programming Modules Explanation**
   1. **Module 1: Data Extraction** (SAS code: N/A)

The first step is to extract the claims of interests from the population(s) of interest, as well as any accompanying demographic or enrollment data.

- - - - 1. Extract population of interest (e.g., all women aged 15-49 years during 2008-2019) from enrollment file.
        2. Extract claims of interest from population of interest. These should be saved to whichever folder will be assigned to the **PGCLAIMS** library.

Use the **Code_m_pgid** code list to identify the **pregnancy-related** **claims of interest** (e.g., all inpatient, outpatient and facility claims with pregnancy-related diagnosis, procedure, or DRG codes) to the subgroup of interest (e.g., women aged 15-49 years). Resulting files might be, for instance, annual files for inpatient, outpatient, and facility header claims, respectively. At a minimum, these must retain the unique ID number for the subject, the service delivery date, and the diagnosis, procedure, and/or DRG codes from that claim.

Use the **Code_ectproc** code list to identify the claims (e.g., all inpatient, outpatient and facility claims) **with an ectopic pregnancy-related procedure** to the subgroup of interest. At a minimum, these must retain the unique ID number for the subject, the service delivery date, and the procedure codes from that claim.

Use the **Code_metho** code list to identify prescription claims **with a methotrexate drug code** among the subgroup of interest. Resulting files might be annual files for prescription claims. At a minimum, these must retain the unique ID number for the subject, the service delivery date, and the drug codes from that claim.

Extract enrollment and demographic data, if using, for any subgroup of interest.

(Note: **Code_preterm_prolong** will be used later)

- - - - 1. Extract all infants (year of birth=service year) with birth year from enrollment file. Save as **PGCLAIM.INFID.**

Extract enrollment data, if using, for infants. Save a file of the unique infant ID numbers for each year of data.

Extract all claims for all infants during year of birth.

(Note: **Code_infant** will be used later)

- 1. **Module 2: Prepare claims into pregnancy records** (Sample SAS code provided)

The second step is to convert the extracted pregnancy-related claims from step 1bi above to ‘pregnancy records’ containing one row per diagnosis, procedure, or DRG code.

- - - - 1. Sample code is provided, but analysts will need to be sure to update to account for the number of diagnosis, procedure, and/or DRG codes in their data files and, if necessary, for multiple claims files. The sample code presumes that the claims files we have the following variables:
- Unique ID for the subject (in SAS code: Enrolid)
- Claim service data (in SAS code: SVCDATE)
- At least one diagnosis (in SAS code: DX), procedure (in SAS code: PROC), or DRG code (in SAS code: DRG)
  - - - 1. In the sample code, the resulting pregnancy records file is called **PGWORK**.**PGWORK** and has one row per code. It contains:
- Unique ID for the subject (in SAS code: Enrolid)
- Claim service data (in SAS code: SVCDATE)
- One diagnosis, procedure, or DRG code (in SAS code: code)
- The associated gestational age (GA), estimated outcome (outcome), code type (code_type), and code version (code_version) applied from **Code_m_pgid** and then retained from the pregnancy identification code set.
  1. **Module 3: Pregnancy Episode Identification** (SAS code provided)

The third step is to identify pregnancy episodes and the primary pregnancy outcome. The resulting dataset is called **PGWORK.PG_S_MI** and has one row per pregnancy episode. It contains:

- Unique subject ID (in SAS code: enrolid)
- Pregnancy number (in SAS code: series)
- Unique ID for the pregnancy episode (in SAS code: newid)
- Primary pregnancy outcome (in SAS code: pgout1)
- Minimum and maximum service dates for the pregnancy episode (in SAS code: svcdate_min, svcdate_max)
- Additional indicator variables for each pregnancy outcome (e.g., sab, lvb, etc.) and outcome/code type combination (e.g., t1_2 indicates a procedure code for live birth) used in later steps.
  1. **Module 4: Pregnancy-Infant Matching** (SAS code provided)

The fourth step is to identify any infants matched to the pregnancy episodes.

- - - - 1. All diagnosis codes from the infant claims dataset are extracted and stored similarly to pregnancy records with one row per diagnosis code. Sample code is provided, but analysts will need to be sure to update to account for the number of diagnosis codes in their data files and for multiple claims files. The resulting file is saved as **PGWORK.** **INF_CLAIM**.
        2. Information is retained on the first claim date (ever) for the infant and the first claim with a code in the **Code_infant** list. The resulting file is saved as **PGWORK.INF_DAY1**.
        3. Infants are matched to pregnancies based on a unique family identifier (in SAS code: famid) and retained as a temporary dataset. A gestational age variable is created from infant codes and saved in a temporary dataset.
        4. The pregnancy-infant(s) matched dataset is saved as **PGWORK.MOTHER_INFANT_PAIR**, with potentially multiple infants per pregnancy. This dataset is used in step 5g below. Among other variables, it contains:
- Unique subject ID for the infant (in SAS code: enrolid_i)
  - - - 1. A final pregnancy-infant verification dataset is saved as **PGWORK.VERIF_INF** with one row per pregnancy (the unique ID number for infants is dropped). It contains:
- Unique subject ID for the mother (in SAS code: enrolid)
- Unique ID for the pregnancy (in SAS code: newid)
- Pregnancy number (in SAS code: series)
- Indicator variable for pregnancy matched to infant (in SAS code: infmatch)
- Number of infants matched to pregnancy (in SAS code: n_inf)
- Infant year of birth (in SAS code: dobyr_i)
- Indicator variable for presence of any infant claim (in SAS code: infclm_all)
- Earliest infant claim service date (in SAS code: infclm_date)
- Indicator variable for and earliest infant claim service date with an infant code (in SAS code: infclm_pg, infclm_pg_date)
- Gestational age estimate from infant codes in pregnancy (in SAS code: ga_inf) and corresponding claim service date (in SAS code: date_inf)
  1. **Module 5: Create Pregnancy Outcome Verification Datasets** (SAS code provided)

The fifth step is to create additional verification datasets. All datasets include, at a minimum:

- Unique subject ID for the mother (in SAS code: enrolid)
- Unique ID for the pregnancy (in SAS code: newid)
- Pregnancy number (in SAS code: series)
  - - - 1. Direct gestational age codes (e.g., Z3A…) are identified in pregnancy episodes by selecting codes in the **Code_m_pgid** list with an outcome value of “GA”. The resulting file is saved as **PGWORK. VERIF_GA6_MI** and contains the direct gestational age estimate and service date/code associated with it (in SAS code: ga_6, date_6, code_6, respectively).
        2. Gestational age estimates associated with each outcome for each pregnancy are identified. The resulting file is saved as **PGWORK.VERIF_GA_OUT_MI** and contains outcome/gestational age/code type variables for the various outcome types included in the pregnancy.
        3. A dataset of verification checks for stillbirths is created. The resulting file is saved as **PGWORK.VERIF_STILLBIRTH** and contains the gestational age estimate and service dates for the stillbirth and livebirth outcomes (in SAS code: ga_sb, ga_lb, date_sb, date_lb, respectively) and difference between stillbirth and live birth service dates (in SAS code: diff_d).
        4. The ectopic procedure and methotrexate prescription files from module 1 are matched to pregnancies. A verification dataset for ectopic pregnancies is saved as **PGWORK.VERIF_ECT** and contains indicator variables for any ectopic procedure and any methotrexate prescription filled during the pregnancy (in SAS code: ectproc, ectmed respectively).
        5. The **code_preterm_prolong** list is used to identify preterm or prolonged pregnancy codes in pregnancies. The resulting file is saved as **PGWORK.** **PRE_PRO_MOTHER.**

The **code_preterm_prolong** list is used to identify preterm or prolonged pregnancy codes in infant claims. The resulting file is saved as **PGWORK.** **PRE_PRO_INFANT.**

The infant and maternal preterm/prolonged pregnancy verification files are combined into one file (**PGWORK.** **VERIF_PP**) that contains the final gestational age estimate based on these codes (in SAS code: ga_pp), among other variables, and indicator variables for whether the code was a direct or indirect identifier of preterm birth/prolonged pregnancy from the maternal or infant record (in SAS code: pp_i_dir, pp_m_dir, pp_i_indir, pp_m_indir).

- 1. **Module 6: Verify and Finalize Pregnancies** (SAS code provided)

The sixth step is to merge the primary pregnancy episodes with all of the verification modules and verify the pregnancy outcome and gestational age. This dataset has one row per pregnancy so potentially multiple rows per woman and contains:

- Unique subject ID (in SAS code: enrolid)
- Pregnancy number (in SAS code: series)
- Unique ID for the pregnancy episode (in SAS code: newid)
- Preliminary pregnancy outcome (in SAS code: pgout1)
- Minimum and maximum service dates for the pregnancy episode (in SAS code: svcdate_min, svcdate_max)
- Number of pregnancy records in pregnancy (in SAS code: n_rcd)
- Matched with infant indicator (in SAS code: infmatch)
- Any hospitalization on infant claim indicator (in SAS code: infclm_pg)
- Date of infant pregnancy-related claim (in SAS code: infclm_pg_date)
- Preterm/prolonged pregnancy indicator (in SAS code: term)
- Source of final outcome (in SAS code: source_out)
- Source of final gestational age (in SAS code: source_ga)
- Delivery date and year (in SAS code: date_out, year_out)
- Gestational age (in SAS code: ga_f)
- Final pregnancy outcome (in SAS code: pgout_f)
- Final last menstrual period date (LMP) (in SAS code: lmp_out)

1. **Algorithm flow**

**
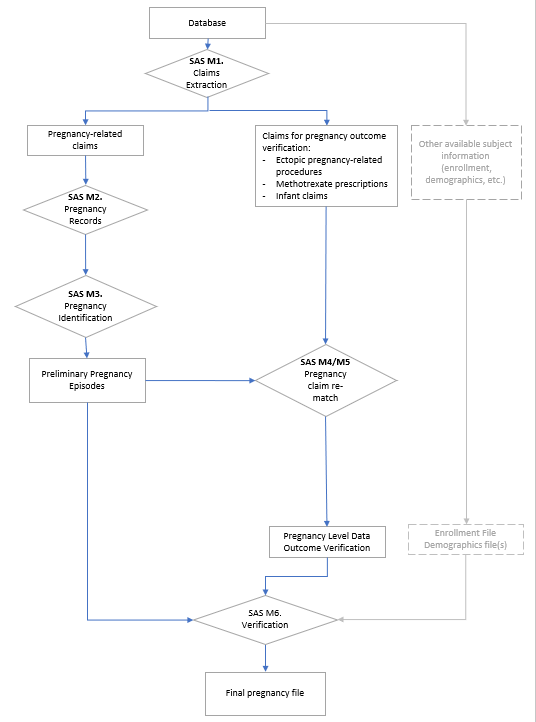
**

1. **SAS Program Summary**

| **Module** | **Contents** |
| --- | --- |
| Module 1: Data extraction | N/A |
| Module 2 : Prepare claims into pregnancy records | Sample code to extract distinct records of Dx/Proc/DRG code and service date for each women |
| Module 3: Pregnancy episode identification | Identification of pregnancy episodes and primary pregnancy outcome. |
| Module 4: Pregnancy-infant matching | Pairing infants to identified pregnancy episodes |
| Module 5: Create pregnancy outcome verification datasets | Extract pregnancy outcome and gestational age codes of each specific type for verification; |
|  | Check all pregnancies with any stillbirth code and verify stillbirths |
|  | Extract all ectopic pregnancy related procedures and Methotrexate Rx that can be matched to pregnancies. |
|  | Extract all preterm or prolonged pregnancy records to verify gestational age estimation. |
| Module 6: Verify and finalize pregnancies | Merge primary pregnancy episodes with multiple verification modules.  Verify pregnancy outcome and gestational age.  Verify enrollment status.  Verify age at the end of pregnancy. |

1. **SAS Program Code**

**************************************************

**************************************************

*Module II. Sample Data Preparation Code

**************************************************

/*Presumes:

- diagnosis codes are saved in variables such as dx

- procedures are saved in in proc

- diagnosis related groups are saved in in DRG

- unique enrollee/subject id is in enrolid

- date of claims is in svcdate

*/

/*Sample code below is for one claims file with one dx, one proc, and one DRG code*/

/*Code will need to be updated to include additional diagnosis, procedure, and/or DRG variables as well as multiple claims files*/

*Set working path;

libname pgclaim ""; /*Save claims files from module 1 here*/

libname pgwork ""; /*Code sets should be saved here*/

*Set codes for pregnancy identification;

**data** newcode;

set pgwork.code_m_pgid;

if code ne "";

**run**;

**proc** **sort** data = /*claim file*/ out = temp nodup;

by enrolid svcdate;

**run**;

*extract diagnosis codes;

**proc** **sql**;

create table temp_dx as

select A.enrolid, A.svcdate, B.code, B.ga, B.code_type, B.outcome, B.code_version

from temp left join newcode B

on A.dx = B.code

where A.dx in (select code from newcode where code_type = "DX");

**quit**;

*extract procedure codes;

**proc** **sql**;

create table temp_pc as

select A.enrolid, A.svcdate, B.code, B.ga, B.code_type, B.outcome, B.code_version

from temp A left join newcode B

on A.proc = B.code

where A.proc in (select code from newcode where code_type = "PROC");

**quit**;

*extract DRG codes;

**proc** **sql**;

create table temp_drg as

select A.enrolid, A.svcdate, B.code, B.ga, B.code_type, B.outcome, B.code_version

from temp A left join newcode B

on put(A.drg, **3.**) = B.code

where put(A.drg, **3.**) in (select code from newcode where code_type = "DRG");

**quit**;

**data** ga_temp;

set temp_pc temp_dx temp_drg;

if enrolid ne **.**;

**run**;

*deduplicate;

**proc** **sort** data = ga_temp nodup out = ga;

by enrolid svcdate;

**run**;

* save permanent file of pregnancy records;

**data** pgwork;

set ga; /*update to include multiple GA datasets, if created*/

if code not in ("");

keep enrolid svcdate code;

**run**;

**proc** **sort** data = pgwork nodup;

by enrolid svcdate code;

**run**;

**data** pgwork.pgwork;

set pgwork;

**run**;

**************************************************

*Module III. Pregnancy Identification

************************************************;

*Set working path;

libname pgclaim "";

libname pgwork ""; /*Code lists saved here*/

*pull in code list to get outcome and gestational age information;

**data** code;

set pgwork.code_m_pgid;

code_mi = substr(note, **1**, **1**);

**run**;

**proc** **sql**;

create table pgwork as

select A.*, B.*

from pgwork.pgwork A left join code B

on A.code = B.code;

**quit**;

*All codes;

**data** pgwork;

set pgwork;

if outcome = "LB+SB" then oc2 = "1.5-LB+SB";

if outcome = "LB" then oc2 = "1-LB";

if outcome = "SB" then oc2 = "2-SB";

if outcome = "SAB" then oc2 = "3-SAB";

if outcome = "IAB" then oc2 = "4-IAB";

if outcome = "ECT" then oc2 = "5-ECT";

if outcome = "GA" then oc2 = "6-GA";

if oc2 = "" then oc2 = "9-ABN";

if code_type = "PROC" then code_type2 = "2-PROC";

if code_type = "DRG" then code_type2 = "3-DRG";

if code_type = "DX" then code_type2 = "1-DX";

**run**;

**proc** **sort** data = pgwork nodup;

by enrolid svcdate code_type2 oc2 ga;

**run**;

*Double check;

/* proc freq data = pgwork; */

/* tables oc2 code_type2 ga; */

/* run; */

**data** pgwork;

set pgwork;

by enrolid svcdate code_type2 oc2 ga;

if oc2 = "4-SAB" then oc2 = "3-SAB";

if oc2 = "3-IAB" then oc2 = "4-IAB";

if oc2 in ("1-LB" "1.5-LB+SB") then lvb = **1**;

if oc2 in ("2-SB" "1.5-LB+SB") then stillbirth = **1**;

if oc2 in ("4-IAB") then iab = **1**;

if oc2 in ("3-SAB") then sab = **1**;

if oc2 in ("5-ECT") then ect = **1**;

if oc2 in ("6-GA") then oth = **1**;

if oc2 in ("9-ABN") then abn = **1**;

if code = "Z3A40" and ga = **39** then ga = **40**;

if oc2 = '1-LB' and code_type2 = '2-PROC' then t1_2 = **1**;

if oc2 = '1-LB' and code_type2 = '3-DRG' then t1_3 = **1**;

if oc2 = '2-SB' and code_type2 = '2-PROC' then t2_2 = **1**;

if oc2 = '3-SAB' and code_type2 = '2-PROC' then t3_2 = **1**;

if oc2 = '3-SAB' and code_type2 = '3-DRG' then t3_3 = **1**;

if oc2 = '4-IAB' and code_type2 = '2-PROC' then t4_2 = **1**;

if oc2 = '4-IAB' and code_type2 = '3-DRG' then t4_3 = **1**;

if oc2 = '5-ECT' and code_type2 = '2-PROC' then t5_2 = **1**;

if oc2 = '5-ECT' and code_type2 = '3-DRG' then t5_3 = **1**;

if oc2 = '1-LB' and code_type2 = '1-DX' then t1_1 = **1**;

if oc2 = '1.5-LB+SB' and code_type2 = '1-DX' then t15_1 = **1**;

if oc2 = '2-SB' and code_type2 = '1-DX' then t2_1 = **1**;

if oc2 = '3-SAB' and code_type2 = '1-DX' then t3_1 = **1**;

if oc2 = '4-IAB' and code_type2 = '1-DX' then t4_1 = **1**;

if oc2 = '5-ECT' and code_type2 = '1-DX' then t5_1 = **1**;

lag_svc = lag(svcdate);

lag_ga = lag(ga);

lag_oc = lag(oc2);

*Inferred LMP, each record;

lmp = svcdate - **7***ga;

*LMP of previous record, interim working variable;

lag_lmp = lag(lmp);

*Difference to previous service date;

dif0 = svcdate - lag_svc;

*Fisrt record;

if first.enrolid then do;

seq = **1**;

series = **1**;

lag_svc = **.**;

day1 = svcdate;*Starting date of episode 1;

dayend = svcdate;

ga_day1 = ga;

out_tag1 = lvb; *Parameter 1, if current or previous pregnancy is livebirth;

end;

retain seq series day1 dayend ga_day1 out_tag1;

if enrolid = lag(enrolid) then do;

*Differnce between inferred LMP and previous record;

dif1 = lmp - lag_svc;

*If LMP is missing, assume a minimal GA for that category;

temp_ga = ga;

if lmp = **.** or (oc2 = "1-LB" and lag_oc ne "6-GA") then do;

if oc2 in ("1-LB" "2-SB") then temp_ga = **20**;

if oc2 in ("3-SAB" "4-IAB" "5-ECT") then temp_ga = **6**;

end;

*Differnce between inferred LMP and previous record;

dif2 = svcdate - **7***temp_ga - lag_svc;

*****************************************************;

*Spilit pontential pregnancy episodes;

if out_tag1 = **1** and (dif0 < **120**

or

(dif1 ne **.** and dif1 < **120** and dif2 < **120**)

or

(dif1 = **.** and dif2 < **120**)

)then do;

seq = seq + **1**;

dayend = svcdate;

end;

else if out_tag1 = **1** and dif0 >= **120**

and (dif1 >= **120** or

(dif0 > **7***temp_ga and dif2 >= **120**))

then do;*Minimum 120 days gap after one livebirth record;

series = series + **1**;*New episode;

seq = **1**;

day1 = svcdate;*Starting date of episode 1;

dayend = svcdate;

ga_day1 = ga;

out_tag1 = lvb;*Update out_tag1 with current code type;

end;

else if out_tag1 = **.** then do;*Minimum 42 days gap after stillbirth and induced abortion;

out_tag1 = lvb;

if (dif0 < **42**

or

(dif1 ne **.** and dif1 < **42**)

or

(dif1 = **.** and dif2 < **42**))

then do;

seq = seq + **1**;

dayend = svcdate;

end;

if dif0 >= **42** and (dif1 >= **42** or

(dif0 > **7***temp_ga and dif2 >= **42**))

then do;

series = series + **1**;

seq = **1**;

day1 = svcdate;

ga_day1 = ga;

out_tag1 = lvb;

end;

end;

end;

*ID for each pregnancy episode;

newid = compress(cat(enrolid, "-", series)," ");

**run**;

*Collaple data by pregnancy episode;

**proc** **sql**;

create table ga3_series as

select enrolid, series, newid, count(*) as n_rcd,

max(lmp) as lmp_max, min(lmp) as lmp_min, max(svcdate) as svcdate_max,

min(svcdate) as svcdate_min, max(lvb) as lvb, max(stillbirth) as stillbirth,

max(iab) as iab, max(sab) as sab, max(ect) as ect,

max(abn) as abn, max(oth) as oth,

max(t1_2) as t1_2, max(t1_3) as t1_3, max(t2_2) as t2_2, max(t3_2) as t3_2,

max(t3_3) as t3_3, max(t4_2) as t4_2, max(t4_3) as t4_3, max(t5_2) as t5_2,

max(t5_3) as t5_3, max(t1_1) as t1_1, max(t15_1) as t15_1, max(t2_1) as t2_1,

max(t3_1) as t3_1, max(t4_1) as t4_1, max(t5_1) as t5_1

from pgwork

group by enrolid, series, newid;

**quit**;

*Step 1.3 Primary pregnancy outcome, round 1;

**data** ga3_series;

set ga3_series;

format pgout1 $10.;

pgout1 = "Unknown";

if lvb = **1** and stillbirth = **1** then pgout1 = "1.5-LB+SB";

else if lvb = **1** then pgout1 = "1-LB"; *live birth;

else if stillbirth = **1** then pgout1 = "2-SB";*Still birth;

else if sab = **1** then pgout1 = "3-SAB";*spontaneous abortion;

else if iab = **1** then pgout1 = "4-IAB";*induced abortion;

else if ect = **1** then pgout1 = "5-ECT";*ecopic pregnancy;

else if oth = **1** then pgout1 = "8-Other";

else if abn = **1** then pgout1 = "9-ABN"; *abnormal;

**run**;

**data** pgwork.pg_s_mi;

set ga3_series;

FORMAT lmp_max lmp_min svcdate_max svcdate_min MMDDYY10.;

**run**;

**************************************************

**************************************************

*Module IV. Prepare infant data for identified pregnancy episodes;

**************************************************

**************************************************

*Set working path;

libname pgclaim "";

libname pgwork "";

***********************************************************************

*Prepare all infant claim from infants claim;

*Convert infant claims to 'records' (one row per diagnosis);

*sample code for one diagnosis code below -- repeat for all diagnosis codes and claims files;

**proc** **sql**;

create table temp_dx as

select distinct enrolid, svcdate, dx as code

from /*infant claims*/

where enrolid ne **.** and dx ne "";

**quit**;

**data** temp_dx;

set temp_dx;

if code ne "";

year = year(svcdate);

**run**;

*Working file for infant claim;

**proc** **sort** data = temp_dx nodup out = pgwork.inf_claim;

by enrolid svcdate code;

**run**;

*Find 1st service date of each infant;

**proc** **sql**;

*Any claim;

create table inf_day1 as

select enrolid as enrolid_i, min(svcdate) as svd_min_i, **1** as clm_all_i

from temp_dx

group by enrolid

order by enrolid;

*Pregnancy related claim;

create table inf_infday1 as

select enrolid as enrolid_i, min(svcdate) as svd_inf_i, **1** as clm_inf_i

from temp_dx

where code in (select code from pgwork.code_infant)

group by enrolid

order by enrolid;

**quit**;

**data** pgwork.inf_day1;

merge inf_day1 inf_infday1;

by enrolid_i;

format svd_min_i svd_inf_i mmddyy10.;

**run**;

******************************************************;

* Combine infants' record for all sources;

*****************************************************;

*Family ID based mother-infant matching;

*All pregnancy episodes;

**proc** **sql**;

create table pg_s as

select enrolid, series, svcdate_max, svcdate_min, pgout1

from pgwork.pg_s_mi;

**quit**;

**data** pg_s;

set pg_s;

newid = compress(cat(enrolid, "-",series), " ");

famid = floor(enrolid/**100**);

year_max = year(svcdate_max);

year_min = year(svcdate_min);

**run**;

*All enrolled infants;

**data** infants;

set /* PGCLAIM.INFID*/

famid = floor(enrolid/**100**);

enrolid_i = enrolid;

dobyr_i = dobyr;

keep enrolid_i famid dobyr_i;

**run**;

**proc** **sql**;

create table infants as

select *, count(*) as n

from infants

group by enrolid_i;

**quit**;

*Matching with Family id and birth year;

**proc** **sql**;

create table m_i as

select A.*, B.*

from pg_s A left join infants B

on A.famid = B.famid and (

A.year_max = B.dobyr_i or A.year_min = B.dobyr_i);

create table i_dup as

select enrolid_i, count(distinct newid) as n_pgm

from m_i

group by enrolid_i;

**quit**;

*Further pair clean;

*keep 1:1 matched mother-infant pairs;

**proc** **sql**;

create table m_i_1 as

select enrolid, series, newid, pgout1, famid, enrolid_i, dobyr_i

from m_i

where enrolid_i in (select enrolid_i from i_dup where n_pgm = **1**);

**quit**;

**proc** **freq** data=m_i_1;

tables pgout1;

**run**;

*3.3 clean data if one infant matched to multiple pregnancies;

**proc** **sql**;

create table m_i_dup as

select enrolid, series, newid, pgout1, famid, enrolid_i, dobyr_i

from m_i

where enrolid_i in (select enrolid_i from i_dup where n_pgm > **1**

and enrolid_i ne **.**);

create table m_i_dup as

select *, min(pgout1) as pgout1_min

from m_i_dup

group by enrolid;

**quit**;

*Keep if primary outcome is LB/LB+SB;

**data** m_i_dup;

set m_i_dup;

if pgout1 in ("1-LB" "1.5-LB+SB");

**run**;

**proc** **sql**;

create table m_i_dup as

select *, count(distinct newid) as n_pgm

from m_i_dup

group by enrolid_i;

create table m_i_dup as

select *, count(distinct enrolid_i) as n_inf

from m_i_dup

group by newid;

**quit**;

**proc** **freq** data = m_i_dup;

tables n_pgm * n_inf/norow nocol nopercent;

**run**;

**data** m_i_dup1;

set m_i_dup;

if n_pgm = **1** and n_inf = **1**;

**run**;

*Multiplet/twins.;

**data** m_i_dup1b;

set m_i_dup;

if n_pgm = **1** and n_inf > **1**;

**run**;

**proc** **sql**;

create table m_i_mlti as

select distinct enrolid, series, newid, n_inf

from m_i_dup1b;

**quit**;

*All infant-mother not 1:1 matched;

**data** m_i_dup2;

set m_i_dup;

if n_pgm > **1**;

**run**;

**proc** **sort** data = m_i_dup2;

by n_inf enrolid series enrolid_i;

**run**;

*Verify duplicated matched infants with 1st claim date;

**proc** **sql**;

create table pg_s_d as

select A.*, B.*

from pg_s A full join m_i_dup2 B

on A.newid = B.newid

where A.newid in (select newid from m_i_dup2);

**quit**;

**proc** **sql**;

create table inf_rec_i as

select A.*, B.*

from pg_s_d A left join pgwork.inf_day1 B

on A.enrolid_i = B.enrolid_i

where A.enrolid_i in (select enrolid_i from pgwork.inf_day1);

**quit**;

**data** inf_rec_i;

set inf_rec_i;

if svd_min_i > svcdate_max + **30** or svd_min_i < svcdate_min - **7** then delete;

**run**;

**proc** **sql**;

create table inf_rec_i as

select *, count(distinct newid) as npgm2

from inf_rec_i

group by enrolid_i;

**quit**;

**proc** **freq** data = inf_rec_i;

tables npgm2;

**run**;

*Total matched mother-infants pairs;

**data** temp_mi;

set inf_rec_i;

if npgm2 = **1**;

**run**;

**data** temp_mi;

set temp_mi m_i_1 m_i_dup1 m_i_dup1b;

**run**;

*Other information from infant records;

*Gestational age in infant records;

**proc** **sql**;

create table infga as

select *

from pgwork.inf_claim

where code in (select code from pgwork.code_infant);

create table infga as

select A.*, B.*

from infga A left join pgwork.code_infant B

on A.code = B.code;

**quit**;

**data** infga;

set infga;

if ga ne **.** and ga > **20**;

**run**;

**proc** **sort** data = infga;

by enrolid svcdate;

**run**;

**data** infga;

set infga;

by enrolid svcdate;

if not first.enrolid then delete;

**run**;

**proc** **sql**;

create table inf_match as

select A.enrolid, A.series, A.newid, A.enrolid_i, A.dobyr_i, B.*

from temp_mi A left join pgwork.inf_day1 B

on A.enrolid_i = B.enrolid_i;

create table inf_match as

select A.*, B.svcdate as date_inf, B.ga as ga_inf

from inf_match A left join infga B

on A.enrolid_i = B.enrolid;

**quit**;

*Final infant-mother pair, one pregnancy episode may match multiple infants;

**data** pgwork.mother_infant_pair;

set inf_match;

**run**;

*****************************************************************************;

* Collapse to pregnancy episode level for final analysis;

**proc** **sql**;

create table pg_inf_match as

select enrolid, newid, series, **1** as infmatch, min(date_inf) as date_inf,

min(ga_inf) as ga_inf, max(ga_inf) as ga_inf_max,

max(clm_all_i) as infclm_all, min(svd_min_i) as infclm_date,

max(clm_inf_i) as infclm_pg, min(svd_inf_i) as infclm_pg_date,

min(dobyr_i) as dobyr_i, count(distinct enrolid_i) as n_inf

from inf_match

group by enrolid, newid, series;

**quit**;

**proc** **freq** data = pg_inf_match;

tables n_inf infclm_all infclm_pg ga_inf ga_inf_max/missing;

**run**;

*Infants at pregnancy level;

**data** pgwork.verif_inf;

set pg_inf_match;

**run**;

**************************************************

**************************************************

*Module V. Pregnancy outcome matching

************************************************;

**************************************************

*Set working path;

libname pgclaim "";

libname pgwork "";

*5.1 Direct gestational age records;

*Extract all direct gestational age codes;

**proc** **sql**;

create table ga_gacode as

select A.enrolid, A.svcdate, A.code,

B.newid, B.series, B.svcdate_max, B.svcdate_min, B.pgout1

from pgwork.pgwork A left join pgwork.pg_s_mi B

on A.enrolid = B.enrolid

where A.code in (select code from pgwork.code_m_pgid where outcome = "GA");

create table ga_gacode as

select A.*, B.outcome, B.ga

from ga_gacode A left join pgwork.code_m_pgid B

on A.code = B.code;

**quit**;

**data** ga_gacode;

set ga_gacode;

if svcdate_min <= svcdate <= svcdate_max;

**run**;

**proc** **sql**;

create table ga_gacode2 as

select enrolid, series, newid, code as code_6,

ga as ga_6, min(svcdate) as date_6

from ga_gacode

group by enrolid, series, newid, code, ga;

**quit**;

**proc** **sort** data = ga_gacode2 nodup;

by newid date_6;

**run**;

**data** pgwork.verif_ga6_mi;

set ga_gacode2;

by newid date_6;

if not last.newid then delete;

format date_6 mmddyy10.;

**run**;

*5.2 All recorded pregnancy outcomes.;

**proc** **sql**;

create table pgwork as

select A.*, B.outcome, B.ga, B.code_type

from pgwork.pgwork A left join pgwork.code_m_pgid B

on A.code = B.code

where A.code in (select code from pgwork.code_m_pgid

where outcome not in ("GA" "ABN"))

and A.code not in ("");

create table pgwork as

select A.*, B.newid, B.series, B.svcdate_max, B.svcdate_min, B.pgout1

from pgwork A left join pgwork.pg_s_mi B

on A.enrolid = B.enrolid;

**quit**;

*All codes;

**data** pgwork;

set pgwork;

if svcdate_min <= svcdate <= svcdate_max;

if outcome = "LB+SB" then oc2 = "1.5-LB+SB";

if outcome = "LB" then oc2 = "1-LB";

if outcome = "SB" then oc2 = "2-SB";

if outcome = "SAB" then oc2 = "3-SAB";

if outcome = "IAB" then oc2 = "4-IAB";

if outcome = "ECT" then oc2 = "5-ECT";

if code_type = "PROC" then code_type2 = "2-PROC";

if code_type = "DRG" then code_type2 = "3-DRG";

if code_type = "DX" then code_type2 = "1-DX";

**run**;

**proc** **sql**;

create table ga_outcome as

select enrolid, series, newid, code_type2, oc2, ga, min(svcdate) as date_o

from pgwork

group by enrolid, series, newid, code_type2, oc2, ga

order by newid, code_type2, oc2;

**quit**;

*Find the first service record/ first outcome-based gestational age record for each type of pregnancy outcome;

**data** ga_outcome;

set ga_outcome;

newid2 = compress(cat(newid, "-", oc2));

**run**;

**proc** **sort** data = ga_outcome nodup;

by newid2 code_type2 date_o;

**run**;

**data** ga_outcome;

set ga_outcome;

by newid2 code_type2 date_o;

f1 = **0**;

ga1 = **0**;

ga_se = **0**;

if first.newid2 then do;

f1 = **1**;

if ga ne **.** then do;

ga1 = **1**;

ga_se = **1**;

end;

end;

retain ga_se;

if newid2 = lag(newid2) and lag(ga) = **.** and ga ne **.** and ga_se = **0** then do;

ga1 = **1**;

ga_se = **1**;

end;

**run**;

**data** ga_outcome;

set ga_outcome;

if ga1 = **1** or f1 = **1**;

**run**;

**proc** **sql**;

create table ga_outcome as

select *, count(*) as n

from ga_outcome

group by newid;

**quit**;

*Pregnancies with claims of the same outcome, and 1st claim has GA;

**proc** **sql**;

create table ga_out1 as

select * from ga_outcome

where n = **1** or

n >= **2** and newid not in (select newid from ga_outcome where f1 = **1** and ga1 = **0**)

order by newid, code_type2, oc2, date_o;

**quit**;

**data** ga_out1;

set ga_out1;

by newid code_type2 oc2 date_o;

if not first.newid then delete;

**run**;

*Pregnancies with 1st claim of one type outcome missing in GA;

**proc** **sql**;

create table ga_out2 as

select * from ga_outcome

where n >= **2** and

newid in (select newid from ga_outcome where f1 = **1** and ga1 = **0**)

order by newid, code_type2, oc2, date_o;

**quit**;

**data** ga_out2a;

set ga_out2;

by newid code_type2 oc2 date_o;

if not first.newid then delete;

**run**;

**data** ga_out2b;

set ga_out2;

by newid code_type2 oc2 date_o;

if first.newid then delete;

**run**;

**proc** **sql**;

create table ga_out2b as

select *

from ga_out2b

where newid in (select newid from ga_out2a where ga = **.**)

and ga ne **.**

order by newid, code_type2, oc2, date_o;

**quit**;

**data** ga_out2b;

set ga_out2b;

by newid code_type2 oc2 date_o;

if not first.newid then delete;

**run**;

**proc** **sql**;

create table ga_out2a as

select A.*, B.ga as ga_o2, B.date_o as date_o2, B.oc2 as oc2_o2,

B.code_type2 as codetype_o2

from ga_out2a A left join ga_out2b B

on A.newid = B.newid;

**quit**;

*First claim of primary outcome as back up;

**data** ga_out3;

set ga_outcome;

if ga ne **.** and oc2 ne "6-GA";

**run**;

**proc** **sort** data = ga_out3;

by newid oc2 code_type2 date_o ga;

**run**;

**data** ga_out3;

set ga_out3;

by newid oc2 code_type2 date_o;

if not first.newid then delete;

**run**;

*************************************;

*Combine all outcome records;

**data** ga_out_all;

set ga_out1 ga_out2a;

**run**;

**proc** **sql**;

create table ga_out_all as

select A.*, B.date_o as date_o3, B.ga as ga_o3,

B.oc2 as oc2_o3, B.code_type2 as codetype_o3

from ga_out_all A left join ga_out3 B

on A.newid = B.newid;

create table pgwork.verif_ga_out_mi as

select *, count(*) as n2

from ga_out_all

group by newid;

**quit**;

*****************************************************************;

*5.3 Further data clean for stillbirths;

*Find all episodes with stillbirth claim;

**proc** **sql**;

create table stillbirth_ck as

select A.enrolid, A.svcdate, A.code,

B.newid, B.series, B.svcdate_max, B.svcdate_min, B.pgout1

from pgwork.pgwork A left join pgwork.pg_s_mi B

on A.enrolid = B.enrolid

where A.enrolid in (select enrolid from pgwork.pg_s_mi

where stillbirth = **1**);

create table stillbirth_ck as

select A.*, B.outcome, B.ga

from stillbirth_ck A left join pgwork.code_m_pgid B

on A.code = B.code;

**quit**;

**data** stillbirth_ck;

set stillbirth_ck;

if svcdate_min <= svcdate <= svcdate_max;

if outcome = "LB+SB" then oc2 = "1.5-LB+SB";

if outcome = "LB" then oc2 = "1-LB";

if outcome = "SB" then oc2 = "2-SB";

if outcome = "SAB" then oc2 = "3-SAB";

if outcome = "IAB" then oc2 = "4-IAB";

if outcome = "ECT" then oc2 = "5-ECT";

if outcome = "GA" then oc2 = "6-GA";

if oc2 = "" then oc2 = "9-ABN";

newid2 = compress(cat(newid, "-", oc2)," ");

**run**;

**proc** **sort** data = stillbirth_ck nodup;

by newid2 svcdate;

**run**;

**proc** **sql**;

create table sck1 as

select *

from stillbirth_ck

where ga ne **.** and oc2 in ("1-LB" "1.5-LB+SB" "2-SB")

order by newid2, svcdate;

**quit**;

**data** sck1;

set sck1;

by newid2 svcdate;

if not first.newid2 then delete;

**run**;

**proc** **sql**;

create table sb1 as

select enrolid, series, newid, min(svcdate) as date_sb, min(ga) as ga_sb

from sck1

where oc2 in ("2-SB" "1.5-LB+SB")

group by enrolid, series, newid

order by enrolid, series, newid;

create table sb2 as

select enrolid, series, newid, min(svcdate) as date_lb, min(ga) as ga_lb

from sck1

where oc2 in ("1-LB" "1.5-LB+SB")

group by enrolid, series, newid

order by enrolid, series, newid;

create table verif_stillbirth as

select A.*, B.*

from sb1 A left join sb2 B

on A.enrolid = B.enrolid and A.series = B.series;

**quit**;

**data** verif_stillbirth;

set verif_stillbirth;

format date_sb date_lb mmddyy10.;

dif_d = date_sb - date_lb;

**run**;

**data** pgwork.verif_stillbirth;

set verif_stillbirth;

**run**;

************************************************************;

*5.4 Further clean for ectopic pregnancy procedure/Methotrexate records;

**data** ectmed;

set /*methotrexate claims dataset(s)*/;

**run**;

*Keep unique ID and service data and dummy variable for presence of methotrexate code called ect_med;

**proc** **sql**;

create table pgwork.ect_med as

select distinct enrolid, svcdate, **1** as ectmed

from ectmed

where enrolid ne **.** ;

**quit**;

* Call in dataset of ectopic procedure claims.;

**data** ect_proc;

set /*ectopic procedure claims dataset(s)*/;

**run**;

*Keep unique ID and service data and dummy variable for presence of ectopic procedure code called ect_proc;

**proc** **sort** data = ect_proc nodup;

by enrolid svcdate;

**run**;

**data** pgwork.ect_proc;

set ect_proc;

ectproc = **1**;

**run**;

**proc** **sql**;

create table verif_ectmed as

select A.enrolid, A.newid, A.series, A.svcdate_max, A.svcdate_min,

B.svcdate as dt1, B.ectmed

from pgwork.pg_s_mi A left join pgwork.ect_med B

on A.enrolid = B.enrolid

where A.enrolid in (select enrolid from pgwork.ect_med);

create table verif_ectproc as

select A.enrolid, A.newid, A.series, A.svcdate_max, A.svcdate_min,

B.svcdate as dt1, B.ectproc

from pgwork.pg_s_mi A left join pgwork.ect_proc B

on A.enrolid = B.enrolid

where A.enrolid in (select enrolid from pgwork.ect_proc);

**quit**;

**data** verif_ectmed;

set verif_ectmed;

if svcdate_min <= dt1 <= svcdate_max;

**run**;

**data** verif_ectproc;

set verif_ectproc;

if svcdate_min <= dt1 <= svcdate_max;

**run**;

**proc** **sql**;

create table v_ectmed as

select enrolid, newid, min(dt1) as dt_ectmed, **1** as ectmed

from verif_ectmed

group by enrolid, newid;

create table v_ectproc as

select enrolid, newid, min(dt1) as dt_ectproc, **1** as ectproc

from verif_ectproc

group by enrolid, newid;

**quit**;

**proc** **sort** data = v_ectmed;

by newid;

**run**;

**proc** **sort** data = v_ectproc;

by newid;

**run**;

*Combine procedure and Rx data;

**data** pgwork.verif_ect;

merge v_ectmed v_ectproc;

by newid;

**run**;

************************************************************;

*5.5 Further clean preterm births and prolonged pregnancies;

*Preterm-prolong term code in mothers' record;

**proc** **sql**;

create table pre_pro as

select A.enrolid, A.svcdate, B.code, B.ga, B.m_i, B.term, B.term_code_type as ptp

from pgwork.pgwork A left join pgwork.code_preterm_prolong B

on A.code = B.code

where A.code in (select code from pgwork.code_preterm_prolong);

create table pre_pro as

select A.*, B.newid, B.series, B.svcdate_max, B.svcdate_min, B.pgout1

from pre_pro A left join pgwork.pg_s_mi B

on A.enrolid = B.enrolid;

**quit**;

*Keep matched claims;

**data** pre_pro;

set pre_pro;

if svcdate_min -**7** <= svcdate <= svcdate_max +**30**;

**run**;

**proc** **sql**;

create table pre_pro as

select A.*, B.*

from pre_pro A left join pgwork.code_preterm_prolong B

on A.code = B.code;

**quit**;

**data** pre_pro;

set pre_pro;

pp_dir = **0**;

if term_code_type = "direct" then pp_dir = **1**;

pp_indir = **0**;

if term_code_type = "indirect" then pp_indir = **1**;

**run**;

*Select max/min ga of each type;

**proc** **sql**;

create table pp_m as

select enrolid, newid, series, max(term) as term, max(ga) as ga_max, min(ga) as ga_min,

max(pp_dir) as pp_m_dir, max(pp_indir) as pp_m_indir

from pre_pro

group by enrolid, newid, series;

**quit**;

*Interim file for mothers;

**data** pgwork.pre_pro_mother;

set pp_m;

**run**;

*Preterm-prolong term in infants' record;

**proc** **sql**;

create table pre_pro_i as

select A.enrolid as enrolid_i, A.svcdate, B.code, B.ga, B.m_i, B.term, B.term_code_type as ptp

from pgwork.inf_claim A left join pgwork.code_preterm_prolong B

on A.code = B.code

where A.code in (select code from pgwork.code_preterm_prolong)

and A.enrolid in (select enrolid_i from pgwork.mother_infant_pair);

create table pre_pro_i as

select A.*, B.*

from pre_pro_i A left join pgwork.mother_infant_pair B

on A.enrolid_i = B.enrolid_i;

create table pre_pro_i as

select A.*, B.newid, B.series, B.svcdate_max, B.svcdate_min, B.pgout1

from pre_pro_i A left join pgwork.pg_s_mi B

on A.enrolid = B.enrolid;

**quit**;

*Keep matched claims;

**data** pre_pro_i;

set pre_pro_i;

if svcdate_min -**7** <= svcdate <= svcdate_max +**30**;

pp_dir = **0**;

if ptp = "direct" then pp_dir = **1**;

pp_indir = **0**;

if ptp = "indirect" then pp_indir = **1**;

**run**;

*Select max/min ga of each type;

**proc** **sql**;

create table pp_i0 as

select newid, series, max(term) as term, max(ga) as ga_max, min(ga) as ga_min,

max(pp_dir) as pp_i_dir, max(pp_indir) as pp_i_indir, count(distinct enrolid_i) as n_inf

from pre_pro_i

group by newid, series;

**quit**;

*Interim file for infants;

**data** pgwork.pre_pro_infant;

set pp_i0;

**run**;

*Create combined preterm-prolong work data;

**proc** **sql**;

create table verif_pp as

select A.enrolid, A.series, A.newid,

B.term as term_m, B.pp_m_dir, B.pp_m_indir,

B.ga_max as ga_p_max_m, B.ga_min as ga_p_min_m

from pgwork.pg_s_mi A left join pgwork.pre_pro_mother B

on A.newid = B.newid

where A.newid in (select newid from pgwork.pre_pro_mother)

or A.newid in (select newid from pgwork.pre_pro_infant);

create table verif_pp as

select A.*, B.term as term_i, B.pp_i_dir, B.pp_i_indir,

B.ga_max as ga_p_max_i, B.ga_min as ga_p_min_i

from verif_pp A left join pgwork.pre_pro_infant B

on A.newid = B.newid;

**quit**;

**data** verif_pp;

set verif_pp;

term = term_i;

if term = "" then term = term_m;

if pp_i_dir = **1** then do;

ga_pp = ga_p_min_i;

term = term_i;

end;

else if pp_m_dir = **1** then do;

ga_pp = ga_p_min_m;

term = term_m;

end;

else if pp_i_indir = **1** then do;

ga_pp = ga_p_min_i;

term = term_i;

end;

else if pp_m_indir = **1** then do;

ga_pp = ga_p_min_m;

term = term_m;

end;

*If without direct ga record, default 42 wks for prolong preg,

36 wks for preterm;

if term = "Post term" and ga_pp = **.** then ga_pp = **42**;

if term = "Preterm" and ga_pp = **.** then ga_pp = **36**;

**run**;

**data** pgwork.verif_pp;

set verif_pp;

**run**;

**************************************************

**************************************************

*Module VI. Pregnancy outcome verification

************************************************;

**************************************************

*Set working path;

libname pgclaim "";

libname pgwork "";

**********************************************************;

**proc** **sql**;

create table pg_s as

select A.*, B.*

from pgwork.pg_s_mi A left join pgwork.verif_ga6_mi B

on A.enrolid = B.enrolid and A.series = B.series;

create table pg_s as

select A.*, B.*

from pg_s A left join pgwork.verif_ga_out_mi B

on A.enrolid = B.enrolid and A.series = B.series;

create table pg_s as

select A.*, B.*

from pg_s A left join pgwork.verif_ect B

on A.enrolid = B.enrolid and A.newid = B.newid;

create table pg_s as

select A.*, B.*

from pg_s A left join pgwork.verif_inf B

on A.newid = B.newid;

create table pg_s as

select A.*, B.dif_d as dif_sb, B.ga_sb

from pg_s A left join pgwork.verif_stillbirth B

on A.newid = B.newid;

create table pg_s as

select A.*, B.term, B.ga_pp, B.pp_i_dir, B.pp_i_indir, B.pp_m_dir, B.pp_m_indir

from pg_s A left join pgwork.verif_pp B

on A.newid = B.newid;

**quit**;

*Outcome Verification;

*Step 1. Clean some uncertain LB;

**data** pg_s;

set pg_s;

source_out = "M_clm";

source_ga = "M_clm";

pgout1a = "9-Unknown";

*starting from infant matched pregnacy;

if t15_1 = **1** then pgout1a = "1.5-LB+SB";

else if infmatch = **1** then pgout1a = "1-LB";

else if t2_1 = **1** then pgout1a = "2-SB";

else if t1_1 = **1** then pgout1a = "1-LB";

else if t3_1 = **1** then pgout1a = "3-SAB";

else if t4_1 = **1** then pgout1a = "4-IAB";

else if t5_1 = **1** then pgout1a = "5-ECT";

else if t3_3 = **1** then pgout1a = "3-SAB";

else if t4_3 = **1** then pgout1a = "4-IAB";

else if t5_3 = **1** then pgout1a = "5-ECT";

else if t2_2 = **1** then pgout1a = "2-SB";

else if t3_2 = **1** then pgout1a = "3-SAB";

else if t4_2 = **1** then pgout1a = "4-IAB";

else if t5_2 = **1** then pgout1a = "5-ECT";

else if t1_2 = **1** then pgout1a = "1-LB";

else if t1_3 = **1** then pgout1a = "1-LB";

*Using GA codes and outcome data to calculate new GA;

if ga_6 ne **.** then lmp_6 = date_6 - **7***ga_6;

ga_6b = **.**;

pgout2 = pgout1a;

*For LB, require at least one DX/Proc/Infant record;

*If without DX/Proc code for LB, set as UNK_LB;

*If LB only from DRG codes, and with no infant claim record;

if pgout2 = "1-LB" and t1_1 = **.** and t1_2 = **.** and infmatch = **.**

then pgout2 = "UNK_LB";

if pgout2 = "1-LB" then do;

if oc2 = "1-LB" then do;

date_out = date_o;

ga_out = ga_inf;

if ga_out = **.** then ga_out = ga;*Infant GA as 1st choice;

end;

if (date_out = **.** or ga_out = **.**)

and oc2_o2 = "1-LB" then do;

date_out = date_o2;

ga_out = ga_o2;

end;

if (date_out = **.** or ga_out = **.**)

and ga_inf ne **.** then do;

date_out = date_inf;

ga_out = ga_inf;

source_out = "I_clm";

source_ga = "I_clm";

end;

ga_f = ga_out;

*Override with direct GA codes if applicable;

ga_6b = ceil((date_out - lmp_6)/**7**);

if **20** < ga_6b <= **42** then do;

ga_f = ga_6b;

source_ga = "M_ga6";

end;

if ga_f < **20** then ga_f = **.**;

end;

*Step 2. Clean LB+SB and SB;

pgout3 = pgout2;

*Correct some SB codes with missing GA;

*if oc2 = "2-SB" and ga ne . then ga = 30;

*if oc2_o2 = "2-SB" and ga_o2 ne . then ga_o2 = 30;

*if oc2_o3 = "2-SB" and ga_o3 ne . then ga_o3 = 30;

*For LB+SB, require direct LB+SB DX code;

*Move pregnancies with seperate LB and SB records to SB;

if pgout2 in ("1.5-LB+SB" "2-SB" "UNK_LB") then do;

if t15_1 = **1** then pgout3 = "1.5-LB+SB";

else if t2_1 = **1** or t2_2 = **1** then pgout3 = "2-SB";

end;

*Find GA for LB+SB;

if pgout3 = "1.5-LB+SB" then do;

if oc2 in ("1.5-LB+SB" "1-LB") then do;

date_out = date_o;

ga_out = ga;

end;

*Some LB+SB codes doesn't have aggsigned GA, use LB/Infant

record to get approximated date and GA;

if (date_out = **.** or ga_f = **.** or ga_f < **20**)

and oc2_o2 in ("1.5-LB+SB" "1-LB") then do;

date_out = date_o2;

ga_out = ga_o2;

end;

if (date_out = **.** or ga_f = **.** or ga_f < **20**)

and oc2_o3 in ("1.5-LB+SB" "1-LB") then do;

date_out = date_o3;

ga_out = ga_o3;

end;

if (date_out = **.** or ga_f = **.** or ga_f < **20**)

and ga_inf ne **.** then do;

date_out = date_inf;

ga_out = ga_inf;

source_out = "I_clm";

source_ga = "I_clm";

end;

*Override with direct GA codes if applicable;

ga_f = ga_out;

ga_6b = ceil((date_out - lmp_6)/**7**);

if **20** < ga_6b <= **42** then do;

ga_f = ga_6b;

source_ga = "M_ga6";

end;

if ga_f < **20** then ga_f = **.**;

end;

*Step 3. Further Clean SB;

pgout4 = pgout3;

*If cannot find GA for LB+SB, combine with SB for verification;

if pgout4 = "2-SB" then do;

if oc2 = "2-SB" then do;

date_out = date_o;

ga_out = ga_sb;

end;

else if oc2_o2 = "2-SB" then do;

date_out = date_o2;

ga_out = ga_o2;

end;

else if oc2_o3 = "2-SB" then do;

date_out = date_o3;

ga_out = ga_o3;

end;

*Override with direct GA codes if applicable;

ga_f = ga_out;

ga_6b = ceil((date_out - lmp_6)/**7**);

if **20** < ga_6b <= **42** then do;

ga_f = ga_6b;

source_ga = "M_ga6";

end;

if ga_f < **20** then ga_f = **.**;

end;

*Among SB with ga missing;

*without LB-DX, nochange;

*with LB DX before SB --> keep SB, using ga from LB codes as GA;

*if LB DX >= 14 days after SB --> LB;

if pgout4 = "2-SB" and date_out = **.** then do;

date_out = date_o;

ga_out = ga;

if dif_sb ne **.** and oc2 = "1-LB" and dif_sb <= -**14** and t1_1 = **1** then pgout4 = oc2;

end;

*Among SBs with uncertainty;

if pgout4 = "2-SB" and (ga_out = **.** or ga_out < **20** or ga_out > **42**) then ga_out = **.**;

if pgout4 = "2-SB" and (ga_out = **.** or ga_out < **20**) then do;

if t3_1 = **1** or t3_2 = **1** or t3_3 = **1** then pgout4 = "3-SAB";

else if t4_1 = **1** or t4_2 = **1** or t4_3 = **1** then pgout4 = "4-IAB";

else if t5_1 = **1** or t5_2 = **1** or t5_3 = **1** then pgout4 = "5-ECT";

end;

*Step 4. Further Clean SAB;

pgout5 = pgout4;

if pgout4 = "3-SAB" then do;

if oc2 = "3-SAB" then do;

date_out = date_o;

ga_out = ga;

end;

if (date_out = **.** or ga_out = **.** or ga_out > **20**)

and oc2_o2 in ("3-SAB" "4-IAB") then do;

date_out = date_o2;

ga_out = ga_o2;

end;

if (date_out = **.** or ga_out = **.** or ga_out > **20**)

and oc2_o3 in ("3-SAB" "4-IAB") then do;

if pgout5 ne "3-SAB" then pgout5 = oc2_o3;

date_out = date_o3;

ga_out = ga_o3;

end;

if pgout5 = "3-SAB" and ga_out > **20** then ga_out = **.**;

end;

*Step 5. Further Clean IAB;

if pgout5 = "4-IAB" then do;

if oc2 = "4-IAB" then do;

date_out = date_o;

ga_out = ga;

end;

if (date_out = **.** or ga_out = **.** or ga_out > **20** )

and oc2_o2 = "4-IAB" then do;

date_out = date_o2;

ga_out = ga_o2;

end;

if (date_out = **.** or ga_out = **.** or ga_out > **20**)

and oc2_o3 = "4-IAB" then do;

date_out = date_o3;

ga_out = ga_o3;

end;

end;

*Step 6. Further Clean ECT;

pgout6 = pgout5;

if pgout6 = "5-ECT" or (pgout6 = "4-IAB" and ga_out = **.**) then do;

if oc2 = "5-ECT" then do;

pgout6 = oc2;

date_out = date_o;

ga_out = ga;

end;

if (date_out = **.** or ga_out = **.** or ga_out > **8**)

and oc2_o2 = "5-ECT" then do;

pgout6 = oc2_o2;

date_out = date_o2;

ga_out = ga_o2;

end;

if (date_out = **.** or ga_out = **.** or ga_out > **8**)

and oc2_o3 = "5-ECT" then do;

pgout6 = oc2_o3;

date_out = date_o3;

ga_out = ga_o3;

end;

end;

if pgout6 = "5-ECT" and ectproc = **.** and ectmed = **.**

then pgout6 = "UNK_ECT";

*Step 7. Finalize outcome and gestational age;

pgout_f = pgout6;

if pgout6 in ("8-Other" "9-ABN" "UNK_ECT") then pgout_f = "9-Unknown";

if pgout_f in ("3-SAB" "4-IAB" "5-ECT") then do;

ga_f = ga_out;

ga_6b = ceil((date_out - lmp_6)/**7**);

if **6**= < ga_6b <= **20** then do;

ga_f = ga_6b;

source_ga = "M_ga6";

end;

if ga_f > **20** then ga_f = **.**;

end;

*Set to unknown;

if date_out = **.** then pgout_f = "9-Unknown";

*For pregnancies with preterm or prolonged pregnancy codes,

final adjust gestational age;

if term ne "" then do;

ga_f = ga_pp;

source_ga = "pp";

end;

*Final QC for GA and outcome date;

if pgout_f in ("1-LB" "1.5-LB+SB" "2-SB") then do;

if ga_f <= **20** then ga_f = **.**;

if ga_f = **.** and **20** < ga_out <= **42** then ga_f = ga_out;

end;

if pgout_f in ("3-SAB" "4-IAB") then do;

if ga_f > **20** then ga_f = **.**;

if ga_f = **.** and **0** < ga_out <= **20** then ga_f = ga_out;

end;

if pgout_f = "5-ECT" then do;

if ga_f > **8** then ga_f = **.**;

if ga_f = **.** and **0** < ga_out <= **8** then ga_f = ga_out;

end;

if ga_f ne **.** and date_out ne **.** then lmp_out = date_out - **7***ga_f;

if pgout_f = "UNK_ECT" then pgout_f = "9-Unknown";

**run**;

*Output final pregnancy file;

**data** pgwork.pg_final;

set pg_s;

year_out = year(date_out);

keep enrolid series newid n_rcd svcdate_max svcdate_min infmatch infclm_pg infclm_pg_date term source_out source_ga date_out ga_f pgout_f lmp_out year_out;

label

ENROLID="Subject ID"

series="Pregnancy number"

newid="Pregnancy ID"

n_rcd="Number of pregnancy records"

svcdate_max="Service date, max"

svcdate_min="Service date, min"

infmatch="Matched with infant"

infclm_pg="Any hospitalization on infant claim"

infclm_pg_date="Date of infant pregnancy-related claim"

term="Preterm/prolonged pregnancy indicator"

source_out="Source of final outcome"

source_ga="Source of final gestational age"

date_out="Delivery date"

year_out="Delivery year"

ga_f="Gestational age"

pgout_f="Final pregnancy outcome"

lmp_out="Final Last menstrual period date (LMP)"

;

**run**;
