# Supplementary material for: Identification of pregnancies and their outcomes in healthcare claims data, 2008–2019: An algorithm
Source: PLoS One. 2023 Apr 24;18(4):e0284893. doi: 10.1371/journal.pone.0284893 (PMC10124843; doi:10.1371/journal.pone.0284893)
Supplement: S1 Table — (DOCX) [file pone.0284893.s005.docx]

**S4 Table. Initial pregnancy outcome compared to final pregnancy outcome, MarketScan 2008-2019 (N=6,520,768 potential pregnancies)**

|  |  | **Initial pregnancy outcome** | | | | | | |  |
| --- | --- | --- | --- | --- | --- | --- | --- | --- | --- |
|  |  | **Live birth** | **Live birth +**  **stillbirth** | **Stillbirth** | **Spontaneous**  **abortion** | **Induced**  **abortion** | **Ectopic pregnancy** | **Unknown** | **Total** |
| **Final pregnancy outcome, after verification steps** | Livebirth | 4,790,212 | 0 | 167 | 0 | 0 | 0 | 0 | 4,790,379 |
|  | Livebirth + stillbirth | 0 | 6,288 | 0 | 0 | 0 | 0 | 0 | 6,288 |
|  | Stillbirth | 0 | 0 | 34,467 | 0 | 0 | 0 | 0 | 34,467 |
|  | Spontaneous abortion | 0 | 0 | 22,028 | 977,583 | 0 | 0 | 0 | 999,611 |
|  | Induced abortion | 0 | 0 | 538 | 0 | 279,175 | 0 | 0 | 279,713 |
|  | Ectopic pregnancy | 0 | 0 | 22 | 0 | 3 | 42,275 | 0 | 42,300 |
|  | Unknown outcome^a^ (excluded) | 18,550 | 0 | 55 | 126 | 55 | 43,135 | 306,089 | 368,010 |
|  | Total | 4,808,762 | 6,288 | 57,277 | 977,709 | 279,233 | 85,410 | 306,089 | 6,520,768 |

Notes: Shaded cells indicates concordant outcome types

^a^ Includes unobservable/right-censored pregnancy outcomes
