# Supplementary material for: Identification of pregnancies and their outcomes in healthcare claims data, 2008–2019: An algorithm
Source: PLoS One. 2023 Apr 24;18(4):e0284893. doi: 10.1371/journal.pone.0284893 (PMC10124843; doi:10.1371/journal.pone.0284893)
Supplement: S2 Table — (DOCX) [file pone.0284893.s006.docx]

**S6 Table. Frequency of single pregnancy outcome type in pregnancy episodes, by final pregnancy outcome type, MarketScan 2008-2019 (N=5,812,699 pregnancies)**

| **Pregnancy outcome** | **Total** | **Single outcome type^a^** | |
| --- | --- | --- | --- |
|  |  | **N** | **(%)** |
| Live birth | 4,527,737 | 4,443,235 | 98.1 |
| Spontaneous abortion | 940,565 | 764,800 | 81.3 |
| Induced abortion | 263,870 | 256,020 | 97.0 |
| Ectopic pregnancy | 42,272 | 41,489 | 98.1 |
| Stillbirth | 32,362 | 2,953 | 9.1 |
| Live birth + Stillbirth | 5,893 | 3,351 | 56.9 |
| All pregnancies | 5,812,699 | 5,508,735 | 94.8 |

^a^ Except livebirth and stillbirth for which two outcome types were counted.
