# Supplementary material for: Identification of pregnancies and their outcomes in healthcare claims data, 2008–2019: An algorithm
Source: PLoS One. 2023 Apr 24;18(4):e0284893. doi: 10.1371/journal.pone.0284893 (PMC10124843; doi:10.1371/journal.pone.0284893)
Supplement: S3 Table — (DOCX) [file pone.0284893.s007.docx]

**S7 Table. Gestational age distribution of pregnancies estimated to end in a live birth (weighted total: 19,190,432 pregnancies^a^) to National Vital Statistics System^b^ (NVSS) estimates**

| **Birth year** | **MarketScan CCAE Data, Weighted n^a^ (%)** | | | | | | | | | | | **NVSS Data^b^ (%)** | | | | | | | | | |
| --- | --- | --- | --- | --- | --- | --- | --- | --- | --- | --- | --- | --- | --- | --- | --- | --- | --- | --- | --- | --- | --- |
|  | **Weighted**  **Total (N)** | **Preterm (< 37 weeks). %** | | | | | **Term (37-41 weeks), %** | | | | **Post-term (>42 weeks), %** | **Preterm (< 37 weeks)** | | | | | **Term (37-41 weeks)** | | | | **Post-term (>42 weeks)** |
|  |  | **< 28** | **28-31** | **32-33** | **34-36** | **Total** | **37-38** | **39-40** | **41** | **Total** |  | **< 28** | **28-31** | **32-33** | **34-36** | **Total** | **37-38** | **39-40** | **41.0** | **Total** |  |
| **2008** | 1,642,585 | 0.3 | 0.9 | 0.5 | 5.2 | **6.9** | <0.1 | 91.7 | <0.1 | **91.7** | **1.4** | -- | -- | -- | -- | -- | -- | -- | -- | -- | -- |
| **2009** | 1,650,429 | 0.3 | 0.9 | 0.5 | 5.0 | **6.8** | <0.1 | 91.7 | <0.1 | **91.8** | **1.5** | -- | -- | -- | -- | -- | -- | -- | -- | -- | -- |
| **2010** | 1,525,022 | 0.3 | 0.9 | 0.5 | 5.0 | **6.7** | <0.1 | 91.7 | <0.1 | **91.8** | **1.5** | 0.7 | 0.9 | 1.2 | 7.2 | 10.0 | 27.3 | 56.1 | 6.2 | 90.0 | 0.5 |
| **2011** | 1,550,801 | 0.3 | 0.8 | 0.5 | 5.0 | **6.6** | <0.1 | 91.8 | <0.1 | **91.8** | **1.6** | 0.7 | 0.9 | 1.2 | 7.0 | 9.8 | 26.1 | 57.5 | 6.2 | 90.2 | 0.4 |
| **2012** | 1,563,278 | 0.3 | 0.8 | 0.5 | 4.9 | **6.5** | <0.1 | 91.8 | <0.1 | **91.9** | **1.6** | 0.7 | 0.9 | 1.2 | 7.0 | 9.8 | 25.5 | 58.3 | 6.1 | 90.2 | 0.4 |
| **2013** | 1,560,098 | 0.3 | 0.8 | 0.5 | 4.9 | **6.5** | <0.1 | 91.5 | <0.1 | **91.6** | **1.9** | 0.7 | 0.9 | 1.2 | 6.8 | 9.6 | 24.8 | 58.9 | 6.3 | 90.4 | 0.4 |
| **2014** | 1,566,780 | 0.3 | 0.8 | 0.5 | 5.0 | **6.6** | <0.1 | 91.0 | <0.1 | **91.0** | **2.4** | 0.7 | 0.9 | 1.2 | 6.8 | 9.6 | 24.8 | 58.7 | 6.5 | 90.4 | 0.4 |
| **ICD-9 average^c^**  **(2008-2014)** | | **0.3** | **0.8** | **0.5** | **5.0** | **6.7** | **<0.1** | **91.6** | <0.1 | **91.6** | **1.7** | **0.7** | **0.9** | **1.2** | **7.0** | **9.7** | **25.7** | **57.9** | **6.3** | **90.3** | **0.4** |
| **2016** | 1,674,167 | 0.6 | 1.1 | 1.0 | 6.1 | **8.7** | 20.0 | 62.0 | 4.3 | **86.4** | **4.9** | 0.7 | 0.9 | 1.2 | 7.1 | 9.9 | 25.5 | 57.9 | 6.4 | 89.8 | 0.4 |
| **2017** | 1,698,640 | 0.6 | 1.1 | 1.0 | 6.1 | **8.7** | 20.5 | 61.7 | 4.2 | **86.3** | **5.0** | 0.7 | 0.9 | 1.2 | 7.2 | 9.9 | 26.0 | 57.5 | 6.3 | 89.7 | 0.3 |
| **2018** | 1,696,547 | 0.6 | 1.1 | 1.0 | 6.2 | **8.8** | 21.2 | 61.2 | 4.0 | **86.4** | **4.9** | 0.7 | 0.9 | 1.2 | 7.3 | 10.0 | 26.5 | 57.2 | 5.9 | 89.7 | 0.3 |
| **2019** | 1,686,101 | 0.6 | 1.1 | 1.0 | 6.3 | **8.9** | 21.9 | 61.4 | 3.5 | **86.8** | **4.3** | 0.7 | 0.9 | 1.2 | 7.5 | 10.2 | 27.3 | 57.0 | 5.2 | 89.5 | 0.3 |
| **ICD-10 average^c^**  **(2016-2019)** | | **0.6** | **1.1** | **1.0** | **6.2** | **8.8** | **20.9** | **61.6** | **4.0** | **86.5** | **4.8** | **0.7** | **0.9** | **1.2** | **7.3** | **10.0** | **26.3** | **57.4** | **5.9** | **89.7** | **0.3** |

^a^ MarketScan Commercial data weighted to national estimates of those with private insurance

^b^ Estimates presented in “Table 20. Births, by gestational age (weeks): United States, 2010–2019” of Martin JA, Hamilton BE, Osterman MJK, Driscoll AK. Births: Final Data for 2019. National Vital Statistics Reports; vol 70 no 2. Hyattsville, MD: National Center for Health Statistics. 2021.

^c^ ICD-9=International Statistical Classification of Diseases, Ninth Revision, Clinical Modification and Procedure Coding Systems; ICD-10=International Statistical Classification of Diseases, Tenth Revision, Clinical Modification and Procedure Coding Systems. Deliveries from 1/1/2008-12/31/2014 were categorized as “ICD-9” while those from 1/1/2016-12/31/2019 were categorized as “ICD-10”.The transition from ICD-9 to ICD-10 coding occurred in October, 2015 so 2015 data are excluded.
