# Supplementary material for: Identification of pregnancies and their outcomes in healthcare claims data, 2008–2019: An algorithm
Source: PLoS One. 2023 Apr 24;18(4):e0284893. doi: 10.1371/journal.pone.0284893 (PMC10124843; doi:10.1371/journal.pone.0284893)
Supplement: S4 Table — (DOCX) [file pone.0284893.s008.docx]

**S8 Table. Sensitivity analysis comparing estimated gestational age without use of direct gestational age codes to the final algorithm estimated gestational age using direct gestational age codes, by gestational age group and International Statistical Classification of Diseases, Clinical Modification and Procedure Coding System (ICD) era.**

| **ICD-9 Era** | | | | | | | | | | | |
| --- | --- | --- | --- | --- | --- | --- | --- | --- | --- | --- | --- |
|  | **Gestational age without direct codes** | | | | | | | | | |  |
| **Final gestational age** | **8 to 10 weeks** | **11-19 weeks** | **20-23 weeks** | **24-28 weeks** | **29-33 weeks** | **34-36 weeks** | **37-38 weeks** | **39-41 weeks** | **>= 42 weeks** | **Unknown** | **Total** |
| **8 to 10 weeks** | 527 | 55 | 0 | 0 | 0 | 0 | 0 | 0 | 0 | 6 | 588 |
| **11-19 weeks** | 22 | 418 | 0 | 0 | 0 | 0 | 0 | 0 | 0 | 5 | 445 |
| **20-23 weeks** | 0 | 1 | 208 | 15 | 38 | 44 | 0 | 254 | 0 | 5 | 565 |
| **24-28 weeks** | 0 | 0 | 1 | 755 | 92 | 91 | 0 | 448 | 0 | 5 | 1392 |
| **29-33 weeks** | 0 | 0 | 0 | 4 | 2862 | 296 | 0 | 1074 | 0 | 2 | 4238 |
| **34-36 weeks** | 0 | 0 | 7 | 15 | 115 | 8988 | 0 | 7439 | 3 | 38 | 16605 |
| **37-38 weeks** | 0 | 0 | 0 | 0 | 4 | 43 | 0 | 1206 | 0 | 1 | 1254 |
| **39-41 weeks** | 0 | 0 | 0 | 1 | 13 | 446 | 0 | 23951 | 1 | 0 | 24412 |
| **>= 42 weeks** | 0 | 0 | 0 | 0 | 4 | 10 | 0 | 7226 | 615 | 4 | 7859 |
| **Unknown** | 36 | 48 | 0 | 0 | 0 | 0 | 0 | 0 | 0 | 10 | 94 |
| **Total** | 585 | 522 | 216 | 790 | 3128 | 9918 | 0 | 41598 | 619 | 76 | 57452 |
| **ICD-10 Era** | | | | | | | | | | | |
|  | **Gestational age without direct codes** | | | | | | | | | |  |
| **Final gestational age** | **8 to 10 weeks** | **11-19 weeks** | **20-23 weeks** | **24-28 weeks** | **29-33 weeks** | **34-36 weeks** | **37-38 weeks** | **39-41 weeks** | **>= 42 weeks** | **Unknown** | **Total** |
| **8 to 10 weeks** | 49839 | 31199 | 0 | 0 | 0 | 0 | 0 | 0 | 0 | 289 | 81327 |
| **11-19 weeks** | 19653 | 20517 | 0 | 0 | 0 | 0 | 0 | 0 | 0 | 588 | 40758 |
| **20-23 weeks** | 507 | 234 | 343 | 231 | 1001 | 106 |  | 974 | 1 | 106 | 3503 |
| **24-28 weeks** | 0 | 0 | 1 | 2507 | 1552 | 384 | 0 | 2863 | 0 | 59 | 7366 |
| **29-33 weeks** | 0 | 0 | 0 | 10 | 14603 | 3819 | 1 | 6206 | 1 | 37 | 24677 |
| **34-36 weeks** | 0 | 0 | 0 | 3 | 369 | 40206 | 1 | 28188 | 48 | 36 | 68851 |
| **37-38 weeks** | 0 | 0 | 0 | 1 | 237 | 6447 | 11 | 231860 | 0 | 21 | 238577 |
| **39-41 weeks** | 0 | 0 | 1 | 1 | 183 | 896 | 24 | 702880 | 37 | 14 | 704036 |
| **>= 42 weeks** | 0 | 0 | 0 | 0 | 8 | 19 | 3 | 19419 | 28557 | 0 | 48006 |
| **Unknown** | 1436 | 2547 | 0 | 0 | 0 | 0 | 0 | 0 | 0 | 357 | 4340 |
| **Total** | 71435 | 54497 | 345 | 2753 | 17953 | 51877 | 40 | 992390 | 28644 | 1507 | 1221441 |

Notes: Shaded cells indicates concordant outcome types. ICD-9=International Statistical Classification of Diseases, Ninth Revision, Clinical Modification and Procedure Coding Systems; ICD-10=International Statistical Classification of Diseases, Tenth Revision, Clinical Modification and Procedure Coding Systems. Deliveries from 1/1/2008-9/30/2015 were categorized as “ICD-9” while those from 10/1/2015-12/31/2019 were categorized as “ICD-10”.
