# Supplementary material for: Identification of pregnancies and their outcomes in healthcare claims data, 2008–2019: An algorithm
Source: PLoS One. 2023 Apr 24;18(4):e0284893. doi: 10.1371/journal.pone.0284893 (PMC10124843; doi:10.1371/journal.pone.0284893)
Supplement: S5 Table — (DOCX) [file pone.0284893.s009.docx]

**S9 Table. Difference between algorithm-estimated last menstrual period (LMP) and fertility (embryo transfer or insemination) procedure-based LMP estimate,^a^ by pregnancy outcome type and ICD era, among pregnancies with co-occurring assisted reproductive procedures (n=107,870 pregnancies^b^)**

| **Pregnancy outcome type and era** | **N** | **Difference in LMP estimates, days**  **(LMP_Algorith_ - LMP_Fertility Procedure_)** | | |
| --- | --- | --- | --- | --- |
|  |  | **Mean** | **Median** | **Interquartile Range** |
| Livebirths | 78,283 | 1 | 0 | -5 to 7 |
| ICD-9 | 53,634 | 1 | 1 | -6 to 9 |
| ICD-10 | 24,649 | 1 | -1 | -5 to 4 |
| Non-live births | 29,587 | -17 | -21 | -35 to -3 |
| ICD-9 | 21,369 | -17 | -22 | -35 to -3 |
| ICD-10 | 8,218 | -17 | -19 | -34 to -2 |
| All | 107,870 | -4 | -2 | -13 to 6 |

Notes: ICD-9=International Statistical Classification of Diseases, Ninth Revision, Clinical Modification and Procedure Coding Systems; ICD-10=International Statistical Classification of Diseases, Tenth Revision, Clinical Modification and Procedure Coding Systems. Deliveries from 1/1/2008-9/30/2015 were categorized as “ICD-9” while those from 10/1/2015-12/31/2019 were categorized as “ICD-10”.
