# Supplementary material for: Identification of pregnancies and their outcomes in healthcare claims data, 2008–2019: An algorithm
Source: PLoS One. 2023 Apr 24;18(4):e0284893. doi: 10.1371/journal.pone.0284893 (PMC10124843; doi:10.1371/journal.pone.0284893)
Supplement: S1 Fig — (DOCX) [file pone.0284893.s010.docx]

**S10 Fig. Sensitivity analysis: Distribution of difference between algorithm-estimated last menstrual period (LMP) and fertility (embryo transfer or insemination) procedure-based LMP estimate,^a^ among pregnancies estimated to end in a live birth with co-occurring assisted reproductive procedures (n=73,241 pregnancies^b^)**

**
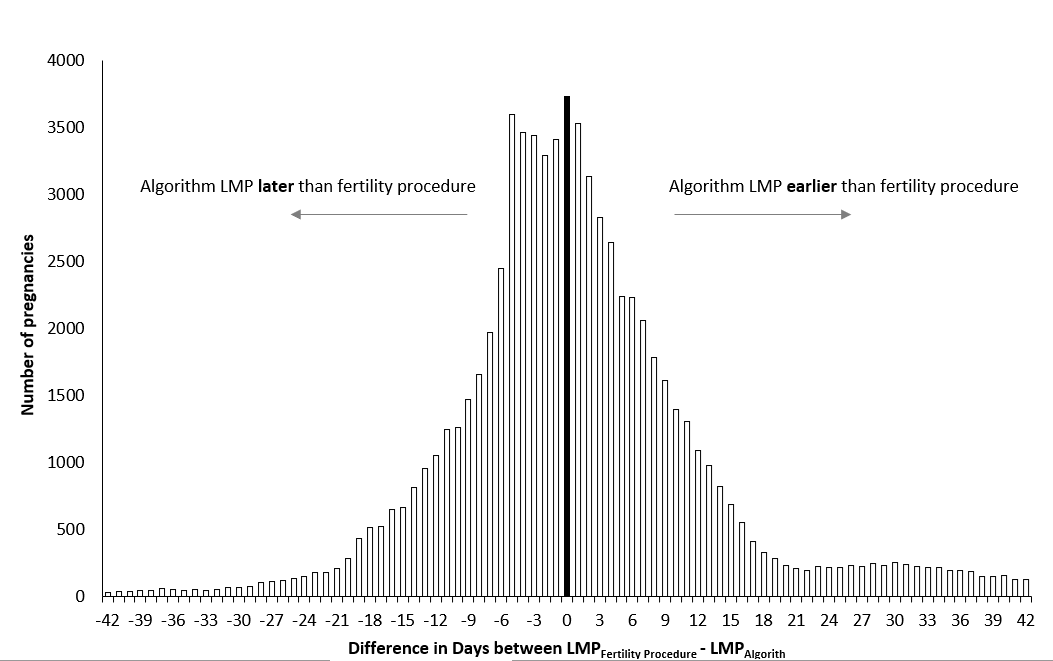
**

Notes: Perfect agreement occurs at 0 days (black bar on graph).

^a^ Defined as fertility procedure date + 14 days.

^b^ Excludes n=5,042 pregnancies with difference < -42 days or >= 42 days between LMPs.
